# The Tumor Milieu Promotes Functional Human Tumor-Resident Plasmacytoid Dendritic Cells in Humanized Mouse Models

**DOI:** 10.3389/fimmu.2020.02082

**Published:** 2020-09-08

**Authors:** Ilona-Petra Maser, Sabine Hoves, Christa Bayer, Gordon Heidkamp, Falk Nimmerjahn, Jan Eckmann, Carola H. Ries

**Affiliations:** ^1^Roche Pharma Research and Early Development, Discovery Oncology, Roche Innovation Center Munich, Penzberg, Germany; ^2^FAU Erlangen, Division of Genetics, Department of Biology, University of Erlangen-Nuremberg, Erlangen, Germany; ^3^Dr. Carola Ries Consulting, Penzberg, Germany

**Keywords:** humanized mice, myeloid-enhanced mice, plasmacytoid dendritic cells (pDC), cancer immunotherapy, human pDC targeting, TLR agonists

## Abstract

Particular interest to harness the innate immune system for cancer immunotherapy is fueled by limitations of immune checkpoint blockade. Plasmacytoid dendritic cells (pDC) are detected in a variety of solid tumors and correlate with poor clinical outcome. Release of type I interferons in response to toll-like-receptor (TLR)7 and TLR9 activation is the pDC hallmark. Mouse and human pDC differ substantially in their biology concerning surface marker expression and cytokine production. Here, we employed humanized mouse models (HIS) to study pDC function. We performed a comprehensive characterization of transgenic, myeloid-enhanced mouse strains (NOG-EXL and NSG-SGM3) expressing human interleukin-3 (hIL-3) and granulocyte-macrophage colony stimulating factor (GM-CSF) using identical humanization protocols. Only in HIS-NOG-EXL mice sufficient pDC infiltration was detectable. Therefore, we selected this strain for subsequent tumor studies. We analyzed pDC frequency in peripheral blood and tumors by comparing HIS-NOG-EXL with HIS-NOG mice bearing three different ovarian and breast tumors. Despite the substantially increased pDC numbers in peripheral blood of HIS-NOG-EXL mice, we detected TLR7/8 agonist responsive and thus functional pDCs only in certain tumor models independent of the mouse strain employed. However, HIS-NOG-EXL mice showed in general a superior humanization phenotype characterized by reconstitution of different myeloid subsets, NK cells and B cells producing physiologic IgG levels. Hence, we provide first evidence that the tumor milieu but not genetically introduced cytokines defines intratumoral (i.t.) frequencies of the rare pDC subset. This study provides model systems to investigate *in vivo* pro- and anti-tumoral human pDC functions.

## Introduction

Efforts to harness the innate immune system for cancer therapy have mainly been focused on macrophages and dendritic cells (DCs). Macrophages are attractive to drug developers because of their abundance in the tumor microenvironment (TME) (1), while the rare dendritic cells are able to present tumor antigen and direct T cell responses (2). Human DCs are classified into three subtypes: Classical CD1c^+^ DCs (cDC2), cross-presenting CD141^+^ DCs (cDC1) and plasmacytoid CD303^+^ DCs (pDC) (3–5). Whereas cDC2 cells play an important role in the stimulation of Th2 and Th17 responses against extracellular pathogens, the cDC1 subset takes up dead cells via CLEC9A and is mainly known to cross-present peptides via MHC class 1 to CD8^+^ T cells (3). The very rare pDCs are capable to produce abundant amounts of all type I interferons in response to stimulation with TLR7 and 9. Hence, they contribute to antiviral immunity (6). While the role of pDCs in chronic viral infections such as HIV or Hepatitis B is well validated (7, 8), their pathogenic role in cancer is less clear. pDCs are detected in a variety of different solid tumors, especially in melanoma, ovarian and breast cancer (5, 9–13). Interestingly, despite their low abundance compared to tumor-associated macrophages (TAMs) or tumor infiltrating lymphocytes, pDC infiltration correlates with poor clinical outcome, likely due to the induction of suppressive immune cells (5, 10–13). However, TLR stimulation of pDCs *in vitro* results in killing of human tumor cells (14). Treatment of in-transit metastases with the TLR7/8 agonist Imiquimod in one melanoma patient showed clinical benefit through pDC activation (15). Therefore, pDCs represent an attractive therapeutic target as restoring their IFN-I-producing function is expected to not only stimulate other immune cells and directly kill tumor cells, but also to reduce the suppressive function of T regulatory cells (Tregs) (12, 13).

The low numbers of these potent cells has hampered development of pDC-targeting strategies, due to the challenging experimental manipulation. Additionally, mouse and human pDCs differ in their biology (8). Mouse pDCs produce high amounts of IL-12, whereas in humans cDCs are the major source for IL-12. Furthermore, TLR7 and TLR9 co-expression is restricted to human pDCs and B cells (8). Functional human pDCs have been successfully reconstituted in different humanized mouse strains and enabled analysis of pDC functionality in virology, autoimmune diseases and in melanoma (13, 15–18).

However, the generation of mice with a human immune system (HIS) represents a quite complex, multi-step process, where experimental parameters for each step can vary between different labs. Immune deficient mice have to be engrafted with human hematopoietic stem cells that requires a pre-conditioning regimen to delete the mouse hematopoietic stem cells (19). As a consequence, humanization protocols deviate in the use of newborn or adult mice, the source of the human cells [hematopoietic stem cells (HSC), fetal liver cells, or peripheral blood mononuclear cells (PBMC) as donor cells], different injections methods as well as irradiation or chemotherapy for pre-conditioning (20, 21). Also animal housing and diet influencing the microbiota have been reported to impact human engraftment quality and quantity (22–25).

Despite the tedious generation process, humanized mice are regularly used as they offer an *in vivo* test system to investigate the heterotypic cross-talk between human tumor cells and human immune cells or to evaluate therapeutic candidates that lack mouse cross-reactivity (19, 26). Although most commonly used HIS mouse models such as HIS-BRG, HIS-NOG or HIS-NSG mice were shown to sufficiently reflect human lymphoid (particularly T and B cell) development, they suffer from some critical limitations. These include impaired lymph node (LN) development, minimal antigen-specific IgG antibody production and lack of antigen-specific human T cell responses (27). Consistently, conventional HIS-mice poorly reconstitute human innate immune cells such as myeloid cells and NK cells, which is attributed to the lack of mouse cytokine cross-reactivity for critical cytokines e.g., IL-3, IL-15, GM-CSF, or M-CSF (28). To overcome this hurdle, transgenic mouse strains were designed to express human IL-3 and GM-CSF, referred to as NOG-EXL and NSG-SGM3 mice (29, 30).

Xenografted human tumors can provide a human cytokine milieu that shapes the functional activity as well as the frequency of tumor-associated myeloid cells. Therefore, we investigated the relative contributions of transgenic and tumor-derived cytokines to myeloid cell and, in particular, pDC reconstitution in both myeloid-enhanced and conventional HIS-models. Importantly, we used identical humanization protocols for all mouse strains, even identical stem cell donors that were pre-selected for high stem cell yield and thus sufficient for all 3 strains. Using two different ovarian tumors and one breast cancer patient derived xenograft (PDX), we identified specific tumors that are capable to generate functional, TLR agonist-responsive pDCs, which do not rely on cytokine expression in transgenic mice nor high pDC numbers in the periphery.

## Materials and Methods

### Ethics Statement

All animal experiments were performed according to the national institutes of health guidelines for the care and use of laboratory animals and European Union directives and guidelines and were approved by the local ethics committees (Regierung von Oberbayern, Munich, Germany).

### Animal Models

Female NOD.Cg-Prkdcscid Il2rgtm1SugTg(SV40/HTLV-IL3,CSF2)10-7Jic/JicTac transgenic mice (NOG-EXL) and non-transgenic NOD.Cg-Prkdcscid Il2rgtm1Sug/JicTac (NOG) mice were purchased from Taconic bioscience (4–5 weeks). Female NOD.Cg-Prkdcscid Il2rgtm1Wjl Tg(CMV-IL3,CSF2,KITLG)1Eav/MloySzJ (NSG-SGM3) were acquired from the Jackson laboratory at the same age. All mouse strains were kept according to the applicable animal protection law in a specific pathogen free (SPF) area. Mice were closely monitored for body weight and general conditions.

### Generation of Humanized Mice

Humanized mice were generated through preconditioning with a chemotherapeutic agent. 15 mg/kg of Busulfan (Busilvex®,Pierre Faber) diluted in 0.9% Saline (Braun) was injected intraperitoneally (i.p.). Twenty-four hours later 10^5^ human CD34^+^ umbilical cord blood-derived HSC (Stem Cell Technologies/Allcells) in 100 μl phosphate-buffered saline (PBS) were transplanted into the mice via intra-venous (i.v.) tail vein injection. Same HSC donors were used and mice were randomized for individual donors.

### Tumor Models

The human ovarian carcinoma cell line OVCAR-5 was purchased from the National Cancer Institute (Cat. Nr. 0507676). The cell line SK-OV-3 is an internal cell line, which was tested and authenticated to be SK-OV-3 after a full match with the reference databases of ATCC, JCRB, RIKEN, KCLB, and DSMZ. All cell lines were confirmed to be free of murine pathogens and murine viruses (Biomedical diagnostics, Hannover, Germany). To generate human xenograft tumors 3 × 10^6^ OVCAR-5 or 5 × 10^6^ SK-OV-3 tumor cells were injected in 100 μl PBS into the right flank of isoflurane anesthetized humanized mice. The human breast cancer patient-derived xenograft BC_038, a triple negative breast cancer, was obtained from Oncotest and transplanted into non-humanized NOG mice for three rounds *in vivo* before use in this study. Tumor fragments were digested with Collagenase D and DNase I (Roche), counted and injected into the mammary fat pad of humanized mice. Tumor growth was monitored twice a week by perpendicular caliper measurement and tumor volume was calculated using the following formula: volume = 0.5 × length^2^ × width.

### TLR Treatment *in vivo*

TLR9 agonist CpG-A (ODN 2216) and TLR7/8 agonist R848 were purchased from InvivoGen. Agonists were diluted with sterile endotoxin free water. Humanized mice were treated with 35 μg/mouse via i.p. or intra tumoral (i.t.) injection. Mice injected with sterile endotoxin containing water served as control.

### Isolation of Human pDCs and *in vitro* Stimulation

Bone marrow (BM)-derived cells as well as splenocytes from humanized mice were pooled from up to ten individual mice to isolate pDCs using a customized isolation kit according to manufacturer's instructions (Stemcell Technologies). Briefly, cells were incubated with normal rat serum, pDC isolation cocktail, biotin and RapidSpheres™. Subsequently, cells were placed in a magnet from Stemcell technologies and the flow-through was collected. Purity of the enriched, untouched pDCs was usually 70%. Human CD303^+^ CD123^+^ cells were used for *in vitro* stimulation using 0.25 μg/μl of TLR agonists for 3 h followed by supernatant collection for cytokine analysis. Splenocytes from all humanized mouse strains did not contain sufficient numbers of pDC required for functional assays.

### Protein Analysis

Cytokine levels in serum and tumor lysates from humanized mice were measured using the Bio-Plex system with Bio-Plex human cytokine 48 or 17-Plex kit as well as single analysis for IFN-α2 according to manufacturer's protocol (Bio-Rad). Chemokine levels in serum and tumor lysates from humanized mice were measured using the Bio-Plex system with human chemokine 40-Plex kit (Bio-Rad). Human and murine FLT3-L were measured by specific ELISA assays (R&D systems).

### Immunoassay for Human-Specific IgG Antibodies

IgG antibodies serum levels of humanized mice were determined by immunoassay (31). Briefly, anti-human Fcγ-pan R10Z8E9 was digoxigenylated and subsequently biotinylated MAB anti-human Fcγ-pan R10Z8E9 was bound to streptavidin-coated microtiter plates at a concentration of 0.5 μg/mL. After incubation for 1 h the unbound antibody was removed by washing. Samples and standards were pre-incubated with 0.05 μg/mL of digoxigenylated MAB anti-human Fcγ-pan R10Z8E9 for 1 h. Afterwords, the mixture was added to wells of microtiter plates coated with the biotinylated anti-human IgG antibodies and incubated for 1 h. After washing, a polyclonal anti-digoxigenin-horseradish peroxidase conjugate (Roche, cat. no. 11633716001) was used to detect the bound digoxigenylated MAB anti-human Fcγ-pan R10Z8E9. A dilution of 50 mU was incubated for 1 h. The HRP of the antibody–enzyme conjugates catalyzed the color reaction of ABTS substrate. A Tecan plate reader at 405 nm wavelength measured the signal at 490 nm.

### Tissue Preparation, Antibodies and Flow Cytometry

Peripheral blood samples were collected into EDTA-coated tubes from the facial vein using lancets. In brief, 20 μl aliquots of whole blood were first lysed with 1X RBC Lysis-buffer (Invitrogen) and washed with MACS running buffer (Miltenyi), before applying the antibodies. Blood leukocytes were tested for human CD45, CD3, CD33, CD14, CD16, CD56, and CD19 (REAfinity/Miltenyi). Live/dead exclusion was performed by propidium iodide (Miltenyi). Eight-color flow cytometry analyses were performed on Miltenyi MACS Quant 10.

The tumor was cut into small pieces using two scalpels. Additionally, tumor mass was simultaneously homogenized in C-tubes by gentleMACS Dissociator from Miltenyi and incubated with Collagenase D and DNase I (Roche). Tumor, BM and spleen were filtered through a combination of filters (100, 70, and 30 μm; Miltenyi) and lysed before samples were blocked for unspecific binding with mouse and human Fc blocking antibodies (Biolegend/Miltenyi). For intracellular staining, cells were fixed for 20 min and permeabilized (Invitrogen) prior to adding the intracellular antibodies for another 20 min.

Splenocytes, BM and tumor samples were tested in different panels for human CD1c, CD3, CD4, CD8, CD11b, CD11c, CD14, CD16, CD19, CD25, CD33, CD40, CD45, CD45RA, CD56, CD62L, CD66b, CD68, CD69, CD83, CD86, CD123, CD141, CD163, CD204, CD206, CD279, CD303, CLEC9A, FOXP3, HLA-DR, and Nkp46 in 18-color combinations (see also Supplementary Table 1). Live/dead staining was performed with fixable ZOMBIE™ UV dye (BioLegend). Antibodies and isotype controls were obtained from BD Bioscience or BioLegend. Eighteen-color flow cytometry analyses were performed on a BD Fortessa device. FCS files were analyzed by FlowJo (Version 10).

### Statistical Analysis

Statistical analyses were performed using Prism 7.0 software (GraphPad Software). Normally distributed data were expressed as mean ± standard error of the means (SEM) unless noted otherwise. Nonparametric data was expressed as median ± interquatile range (IQR) and is indicated in the figure legend as such. The student *t*-test was used to compare normally distributed two-group data. Ordinary one-way ANOVA with Tukey multiple comparison or Kruskal-Wallis ANOVA with Dunn's multiple comparison were performed, if more than two groups were analyzed. Survival curve analysis was achieved using the log-rank (Mantel-Cox) and Gehan-Breslow-Wilcoxon tests. Individual data points are shown if *n* < 5. Each dot represents an individual mouse or analyzed sample. Statistical tests used are indicated in the figure legends.

## Results

### Characterization of Humanized NOG and Myeloid Enhanced Mice

**Figure 1:**
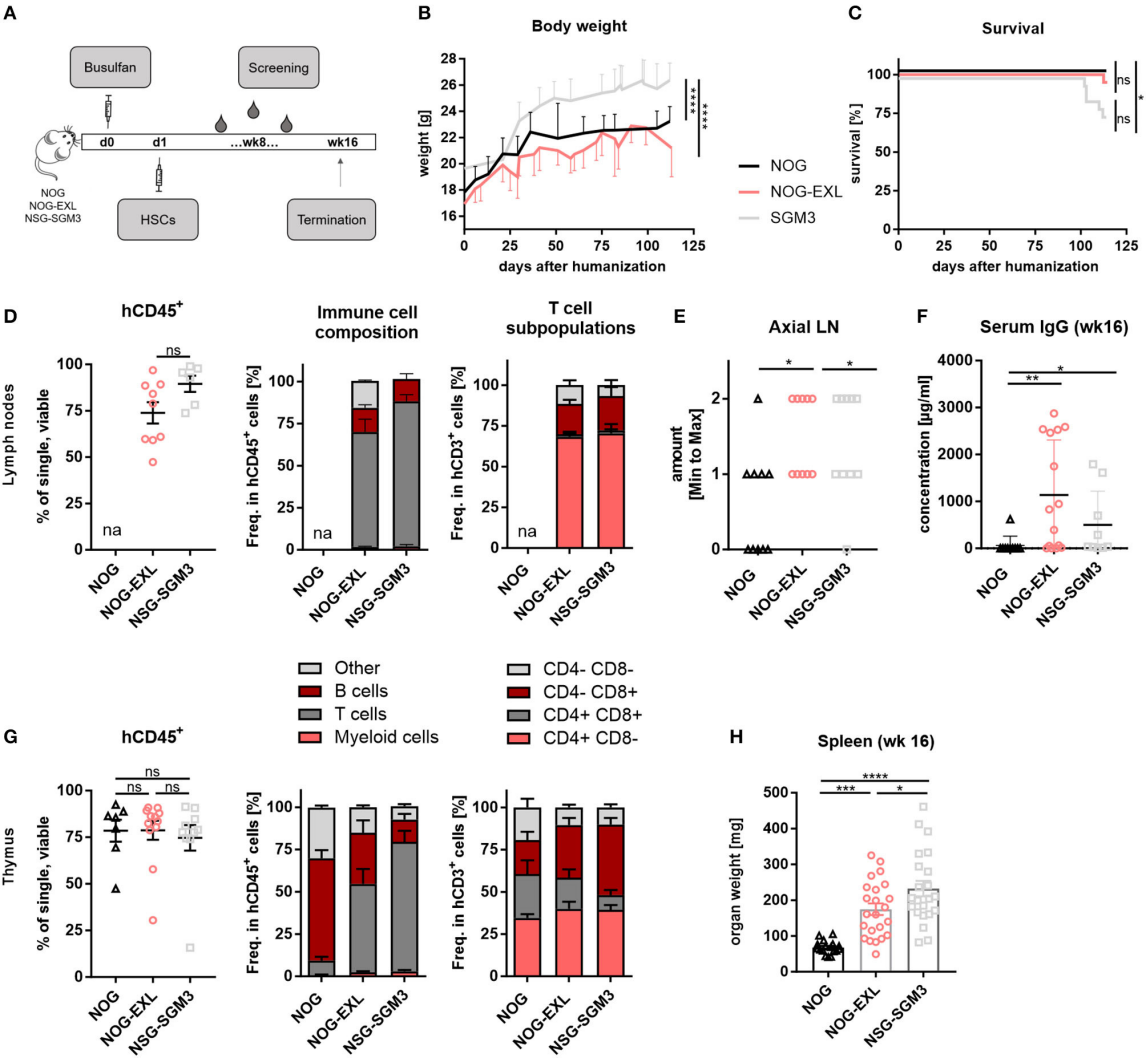
Differences between identically humanized mouse strains in body weight, survival and immune cell composition. **(A)** Reconstitution protocol, graphic overview: NOG, NOG-EXL, and NSG-SGM3 (5–6 weeks) were pretreated with Busulfan and injected with human HSCs. Body weight (BW) and peripheral blood was screened biweekly. At termination (week 16), different peripheral organs were analyzed for human leukocytes. **(B)** BW kinetic: NOG, NOG-EXL, and NSG-SGM3 were monitored from day 0 until termination, *n* = 18–341, one-way ANOVA, Tukey's multiple comparison test. ****p < 0.0001. **(C)** Survival curve comparison, log-rank (Mantel-Cox) or Gehan-Breslow-Wilcoxon test provided same statistical results, error bars indicate mean ± SD, *n* = 20/strain, *p < 0.05. **(D)** Human leukocyte composition (CD45^+^) in lymph nodes (LNs) as percentage, *n* = 6–10, one-way ANOVA, Tukey's multiple comparison test, ****p < 0.0001; Immune cell composition in CD45^+^ and T cells in CD3^+^. huNOG was excluded from analysis due to human CD45 levels < 25%. **(E)** Axial LN amount from all humanized mouse strains analyzed, *n* = 10/strain. One-way ANOVA, Tukey's multiple comparison test. *p < 0.05. **(F)** Total human IgG concentration in serum (week 16), *n* = 10/strain, Kruskal-Wallis ANOVA with Dunn's multiple comparison test, error bars indicate median ± IQR, *p < 0.05; **p < 0.01. **(G)** Human leukocyte composition in thymus as percentage, *n* = 6–10, one-way ANOVA, Tukey's multiple comparison test, ****p < 0.0001, Immune cell composition in CD45+ and T cell subsets. **(H)** Spleen weight in mg (week 16), one-way ANOVA, Tukey's multiple comparison test, *n* = 17–23. *p < 0.05; ***p < 0.001; ****p < 0.0001.

To delineate the contribution of transgene and tumor-derived cytokines to DC development in humanized mice, we first performed a detailed characterization of different myeloid enhanced transgenic mouse strains. For humanization NOG, NOG-EXL and SGM3 mice were pretreated with Busulfan and injected i.v. with human CD34^+^ hematopoietic stem cells (HSCs) at 5–6 weeks of age. Body weight was monitored and humanization status was determined in peripheral blood in 2-week intervals (Figure 1A). Even though all mouse strains were humanized using the same protocol, differences in body weight and survival were observed (Figures 1B,C). HIS-NSG-SGM3 showed significantly increased body weight throughout the study accompanied by decreased overall survival compared to HIS-NOG and HIS-NOG-EXL mice. We detected a high proportion of human CD45^+^ immune cells in LNs of HIS-NOG-EXL and HIS-NSG-SGM3 (Figure 1D) in contrast to the HIS-NOG control strain. Therefore, we omitted LN analysis from the latter due to the low humanization level <25%. Anatomically, only myeloid enhanced mice showed robust axial LN development with HIS-NOG-EXL mice having the largest LNs (Figure 1E, Supplementary Figure 1A). LN immune cell analysis revealed a large T cell population in the transgenic mice, with CD4^+^ T cells being the predominant subset with over 50% (Figure 1D).

One limitation of conventional HIS models is the lack of antibody class switch. Remarkably, myeloid-enhanced HIS mice and here in particular HIS-NOG-EXL mice, showed consistently high levels of total human IgG 16 weeks after humanization (Figure 1F). These levels are comparable with the increased IgG levels of NSG-SGM3 BLT compared to NSG mice and mCD47/BALB-HIS mice and even 2-fold higher than in IL-6 knock-in mice or as in specifically developed BRGST mice (27, 32–34). Furthermore, IgG was detectable from week 10 onward albeit at lower concentration (Supplementary Figure 1B).

Thymus size (Supplementary Figure 1C) as well as human CD45^+^ levels (Figure 1G) were comparable across all strains. Yet again, transgenic mice presented an increased T/B cell ratio. Remarkably, HIS-NOG mice showed a high number of thymic B cells. Since this strain displays in general poor B cell differentiation, manifested in a lack of Ig class switching and absence of serum IgG, we hypothesize that immature B cell precursors may partially accumulate in the thymus.

CD4^+^ T cell frequencies were comparable, while transgenic strains had higher CD8^+^ and lower double positive frequency compared to HIS-NOG (Figure 1G). Splenomegaly was observed in both myeloid enhanced mouse strains compared to HIS-NOG (Figure 1H, Supplementary Figure 1D) that matched in size with other immune competent mouse strains. However, hepatosplenomegaly was exclusively detected in HIS-NSG-SGM3 mice (Supplementary Figures 1E,F), confirming published data (35, 36). Together, our data imply that IL-3 and GM-CSF transgene expression favor CD8^+^ T cell reconstitution and antibody class switch associated with improved LN and thymus development.

### Myeloid Enhanced HIS Mice Demonstrate Superior Overall Human Immune Cell Reconstitution

**Figure 2:**
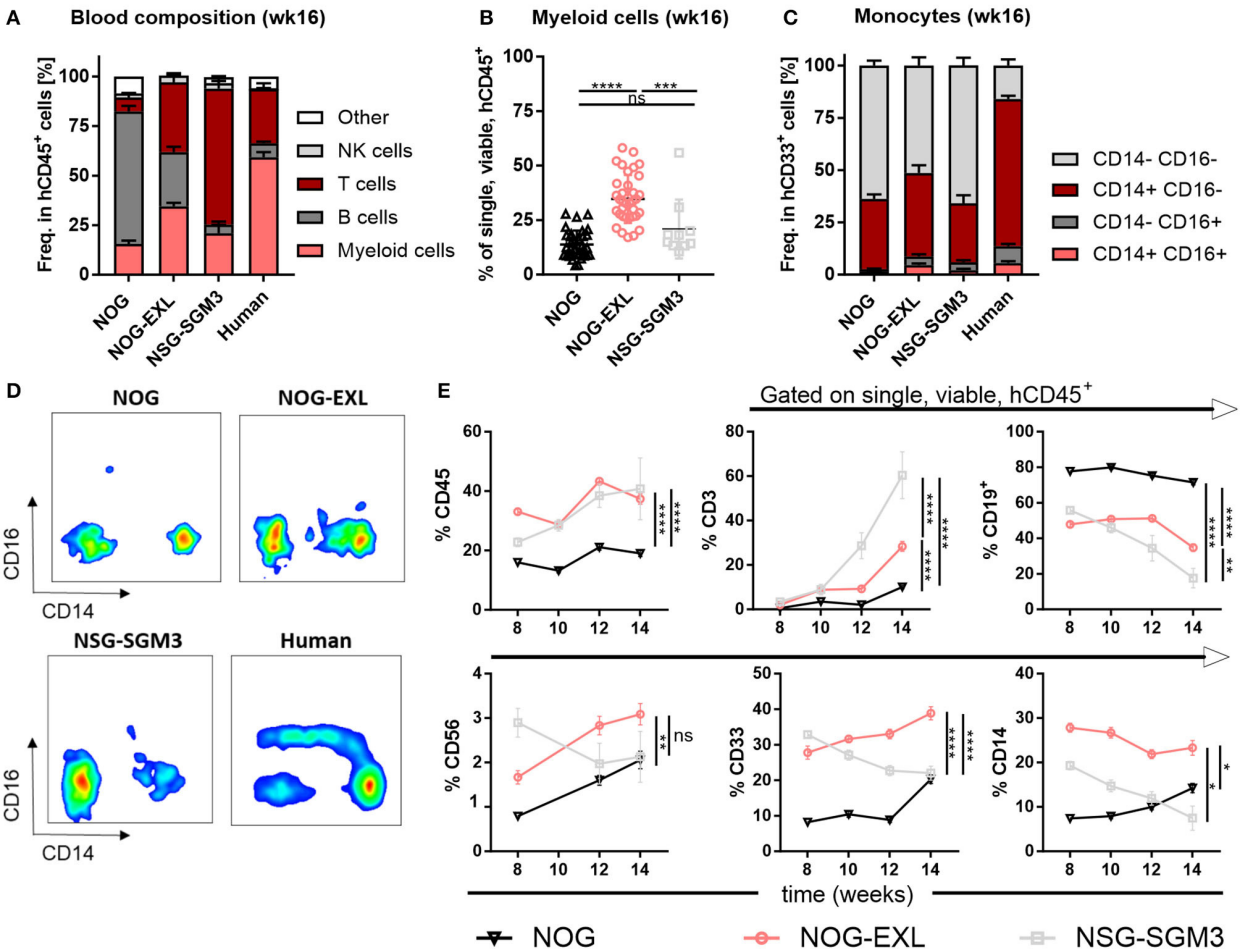
Peripheral blood analysis demonstrates improved human immune cell reconstitution. **(A)** Human leukocytes composition (CD45^+^) in peripheral blood (PB) 16 weeks after reconstitution as percentage, *n* = 28–40. **(B)** Comparison of human myeloid cells (CD33^+^) in human leukocytes (CD45^+^) as percentage from same mice as in A, one-way ANOVA, Tukey's multiple comparison test. *p < 0.05; ***p < 0.0005; ****p < 0.0001, *n* = 10–39. **(C)** Human monocyte composition in total CD33^+^ in PB 16 weeks after reconstitution or in human PBMCs as percentage, *n* = 5–40. **(D)** Representative FACS plots for human monocyte populations (CD16^+/−^/CD14^+/−^ in CD45^+^) in PB of humanized mice in week 16 or in human PBMCs. **(E)** Different immune cell populations (CD45^+^ in single viable; CD19^+^, CD3^+^, CD33^+^, CD56^+^, and CD14^+^ in CD45^+^) in HIS-NOG, HIS-NOG-EXL and HIS-NSG-SGM3 over time (week 8–14), ordinary 2-way ANOVA, *p < 0.05; **p < 0.001; ***p < 0.0005; ****p < 0.0001, *n* = 28–121.

Next, we evaluated the general human immune cell engraftment in peripheral blood of different HIS models by flow cytometry (Supplementary Figure 2A). Whereas, lymphoid and myeloid cell distribution was comparable in blood of HIS-NOG-EXL, B and T cells dominated in HIS-NOG and HIS-NSG-SGM3, respectively (Figure 2A). Amongst the different strains tested, we found that HIS-NOG-EXL mice most closely mimic the human immune cell composition 16 weeks after reconstitution (Figure 2A). The transgene expression led to significantly improved CD33^+^ myeloid reconstitution particularly in HIS-NOG-EXL mice (Figure 2B, Supplementary Figures 2B,C). Additionally, we observed marked differences in the monocytic lineage between humanized mice and human blood characterized by CD14 and CD16 expression. Classical CD14^+^CD16^−^ monocytes were present in blood of all HIS strains, intermediate CD14^+^CD16^dim^ and non-classical CD14^−^/CD16^+^ monocytes were barely detectable (Figures 2C,D). Contrary to human blood, where classical monocytes are the dominant population, humanized mice underrepresent this population at the expense of a high frequency of immature CD33^+^ cells lacking monocyte markers. This phenomenon might be attributed to the missing M-CSF crucial for monocyte/macrophage differentiation (28).

White blood cell composition in HIS mice is known to change over time (29, 30). Humanization was maintained as HIS-NOG-EXL and HIS-NSG-SGM3 displayed significantly higher overall engraftment levels when compared to HIS-NOG (Figure 2E). In contrast, individual immune cell subsets followed different kinetics. Whereas, CD3^+^ T cells slightly increased, CD19^+^ B cells declined over time. Myeloid enhanced HIS mice had higher frequencies of CD56^+^ NK cells at early time points while only HIS-NOG-EXL mice maintained their NK population. Strikingly, increased frequencies of myeloid cells (CD33^+^/CD14^+^) were only observed in HIS-NOG-EXL mice throughout the screening period (Figure 2E). Despite transgene expression for enhanced myeloid reconstitution, HIS-NSG-SGM3 mice displayed decreasing NK-, B and myeloid cell frequencies over time.

**Figure 3:**
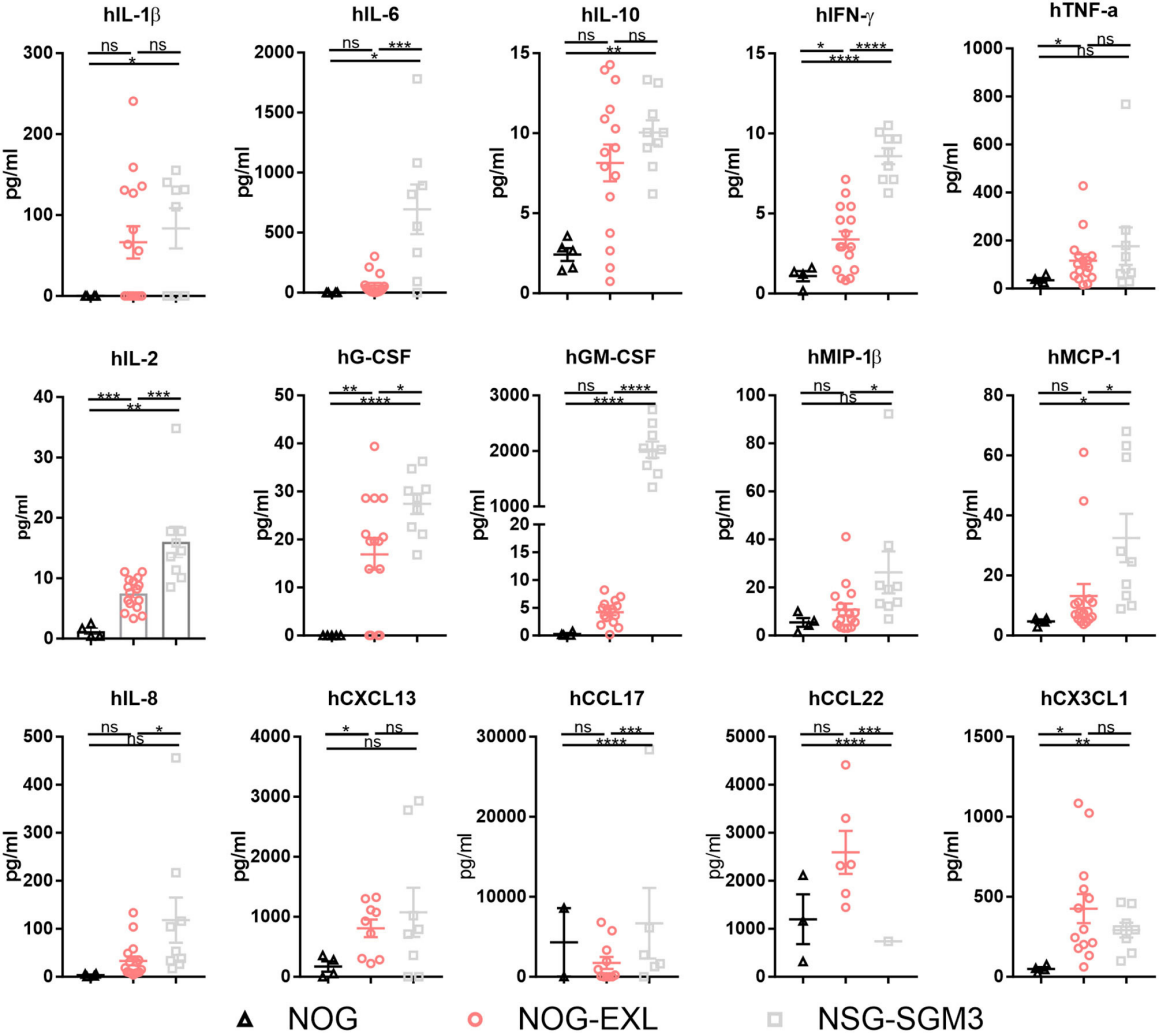
Myeloid-enhanced mice show increased inflammatory profile. Human cytokines and chemokines analysis by Bio-Plex in sera of humanized NOG, NOG-EXL and NSG-SGM3 mice, two-tailed unpaired *t*-test, error bars indicate mean ± SEM, *p < 0.05; **p < 0.005; ***p < 0.0005; ****p < 0.0001; ns, not significant, *n* = 4–16.

Besides cellular immune composition, we were also interested in the human cytokine profile. Both myeloid-enhanced HIS mice showed significantly increased cytokine levels (Figure 3, Supplementary Figure 3). In line with published data (36, 37) we found cytokines/chemokines elevated especially in HIS-NSG-SGM3, such as the pro-inflammatory IL-1β, IL-6, IL-8, TNF-α, IFN-γ, MCP-1, and MIP-1β as well as anti-inflammatory IL-10 (Figure 3). Besides, cytokines and chemokines involved in T cell differentiation, e.g., IL-4, CX3CL1, and CCL17, but also Treg recruitment such as CCL22 were significantly elevated (Figure 3). Moreover, additional proinflammatory molecules showed a trend for upregulation (Supplementary Figure 3). As expected, human GM-CSF serum levels were strongly increased in sera of transgenic mice, in particular of HIS-NSG-SGM3 mice with ~2 ng/ml. Non-humanized mice control sera were used to confirm the specificity of the multiplex assay for human cytokines (data not shown).

Collectively, we confirmed an enhanced human myeloid reconstitution in both mouse strains. However, HIS-NOG-EXL outperform HIS-NSG-SGM3 mice in maintaining long-term human myeloid cell reconstitution together with a more physiologic, low inflammatory cytokine profile.

### HIS-NOG-EXL Mice Develop DC Subsets in Spleen and Have Functional Human pDCs

**Figure 4:**
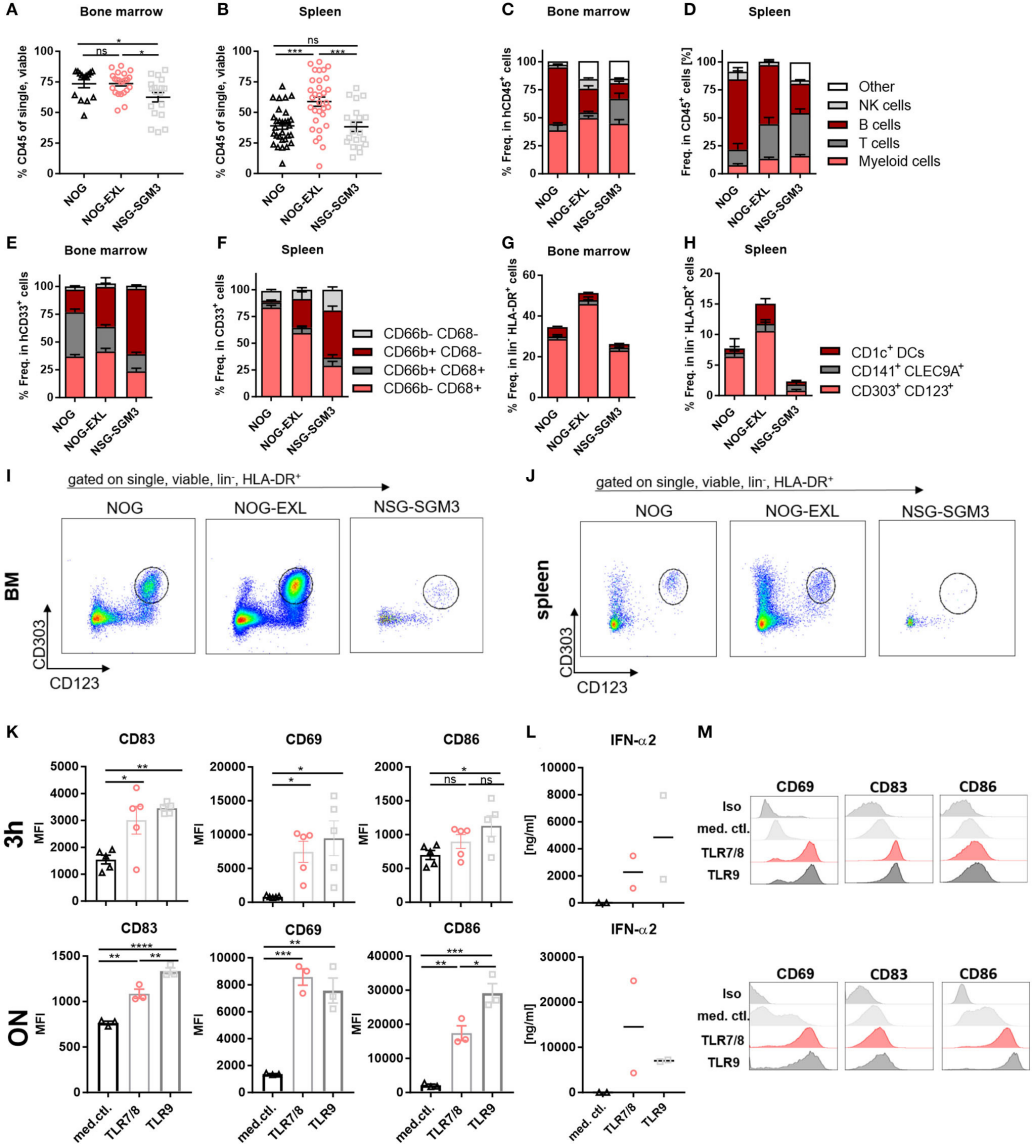
HIS-NOG-EXL mice develop functional human pDCs. **(A)** Human CD45^+^ in BM, ordinary one-way ANOVA, Tukey's multiple comparison test, *p < 0.05, *n* = 14–23. **(B)** Human CD45^+^ comparison in spleens, one-way ANOVA, Tukey's multiple comparison test, *p < 0.05, *n* = 20–32. **(C)** Immune cell composition in CD45^+^ from BM; **(D)** or splenocytes. **(E)** Myeloid cells in CD33^+^ from BM; **(F)** from splenocytes. **(G)** DC subpopulations in HLA-DR^+^ from BM, **(H)** from splenocytes. **(I)** Representative FACS plots of human pDCs (CD303^+^/CD123^+^) gated on single, viable, lineage negative, HLA-DR^+^ in HIS-NOG or HIS-NOG-EXL BM and **(J)** spleen. **(K)** Activation marker MFIs (CD83, CD69, CD86), gated on pDCs (CD123^+^/CD303^+^) enriched from ten pooled HIS-NOG-EXL BM with TLR7/8 or TLR9, duplicates were performed, one-way ANOVA, Tukey's multiple comparison test. *p < 0.05; **p < 0.001; ***p < 0.0005; ****p < 0.0001, *n* = 3/condition. **(L)** IFN-α2 levels in supernatant of TLR-agonist treated or non-treated pDCs enriched from pooled HIS-NOG-EXL BM, measured after 3 h or overnight (ON) stimulation in duplicates. **(M)** Representative activation marker histograms with (TLR7/8 or TLR9) or without stimulation (media control = med. ctl.) ON, gated on lin^−^, HLA-DR^+^, pDC^+^.

Major subsets of myeloid cells undergo terminal differentiation in tissues (38). Following the analysis of immune cell composition in LN and thymus (Figures 1D–H), we subjected spleen and BM samples of the same mice to flow cytometry analysis of human myeloid cells (DCs, macrophages and granulocytes) (Supplementary Figures 4A–C). Contrary to both HIS-NOG strains, HIS-NSG-SGM3 mice showed lower BM humanization, while HIS-NOG-EXL had a high humanization level in spleen (Figures 4A,B). Concomitantly to the increased myeloid cell frequency in transgenic mice, we found increased T and NK cell numbers, while B cells were reduced in both lymphoid organs (Figures 4C,D). Similar to peripheral blood, HIS-NSG-SGM3 exhibited the highest CD3^+^ T cell frequency in BM and spleen. Notably, increased numbers were not only observed for CD4^+^ and CD8^+^, but also Tregs (Supplementary Figures 4D,E), confirming previously published data (29).

Surprisingly, obvious differences were detected within the myeloid compartment of both transgenic GM-CSF and IL-3 HIS-models. While CD66b^−^CD68^+^ myeloid cells were the most abundant subset in BM and spleens of HIS-NOG and HIS-NOG-EXL, CD66b^+^CD68^−^ granulocytes were enriched in HIS-NSG-SGM3 (Figures 4E,F), which might be attributed to their elevated G-CSF levels (35). Of note, CD66b^+^CD68^+^ cells represent a substantial fraction of myeloid cells in BM that is reduced in transgenic HIS mice. We interpret these unusual CD68^+^/CD66b^+^ cells as common myeloid progenitors that retain expression of both markers.

Next, we detected all major subsets of human DCs (CLEC9A^+^ cDC1s, CD1c^+^ cDC2s, and CD303^+^ pDCs) in BM and spleens of all HIS-mice independent of the transgene (Figures 4G–J, Supplementary Figure 4F). In general, higher DC frequencies were found in BM compared to spleens with pDCs being the most abundant subset in both organs. Among the three strains, HIS-NOG-EXL exhibited the highest overall DC level. Notably, only in HIS-NSG-SGM3 DC frequencies correlated between BM and periphery.

Further, we tested the functionality of DC subsets in HIS-NOG and HIS-NOG-EXL mice, as they phenotypically resembled human blood-derived cells. Here we focused on the functional characterization of pDCs in HIS-NOG-EXL mice, due to lower immune cell counts in BM of HIS-NOG and the very low frequency of conventional DCs in general. Induced cytokine release upon TLR activation has been conducted before as reliable readout for pDC function (13, 15). Hence, we cultivated isolated pDCs from pooled BM for 3 h or overnight in presence or absence of TLR agonists. TLR7/8 and TLR9 stimulation of pDCs resulted in increased expression of early activation marker CD69, activation marker CD83 and co-stimulatory molecule CD86. Further, pDCs secreted IFN-α2 upon TLR activation (Figures 4K–M). IFN-α2 levels were maintained and resulted in increased concentrations upon overnight incubation (Figure 4L). Thus, human pDCs isolated from a humanized mouse model are not only reconstituted in sufficient numbers, but are also functional.

### The Tumor Model Dictates Intratumoral Human Immune Cell Composition

**Figure 5:**
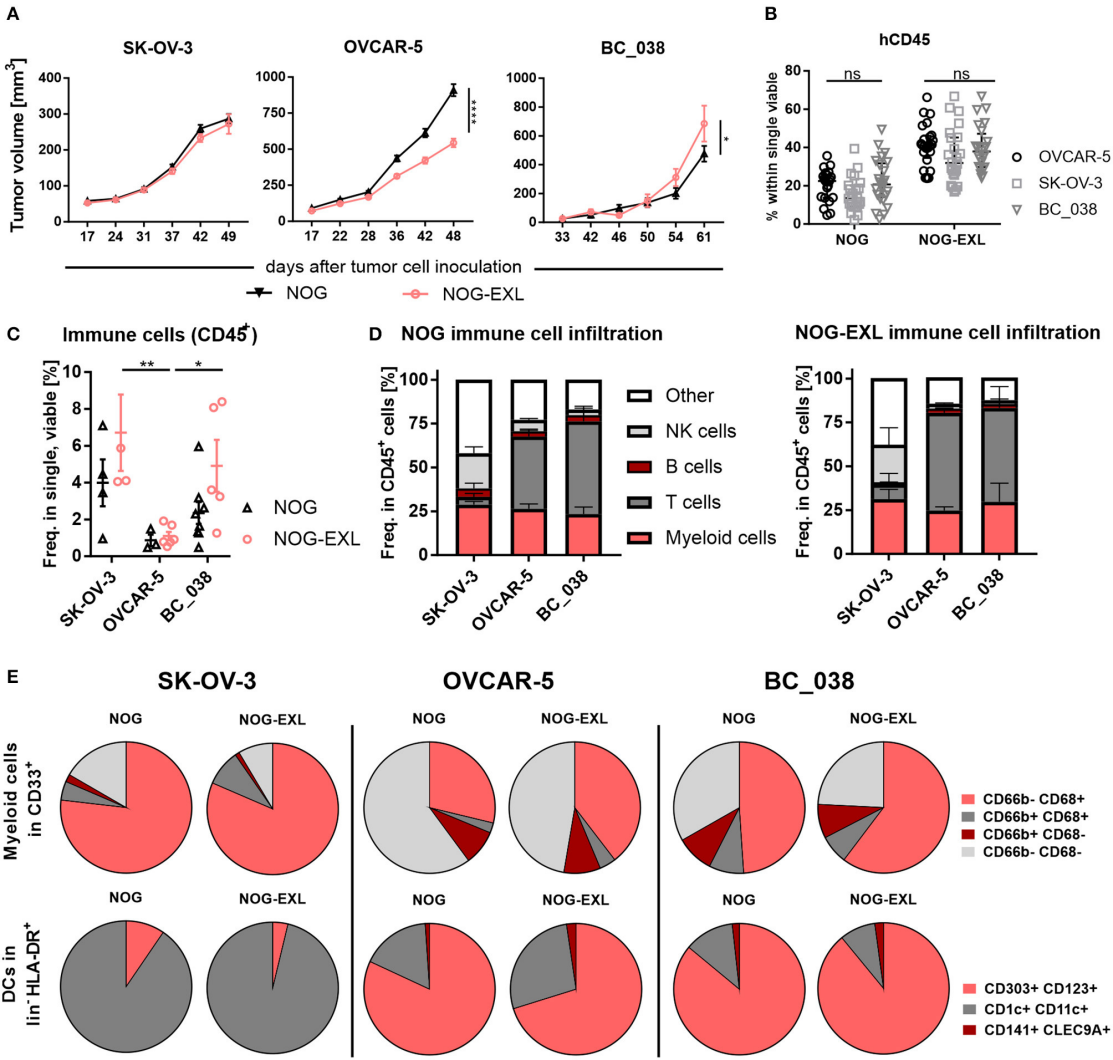
The tumor model dictates intratumoral human immune cell composition. **(A)** Tumor volume [mm^3^±SEM] in humanized mice injected with different tumor models. 2-way ANOVA, *p < 0.05; **p < 0.001; ****p < 0.0001, error bars indicate median ± 95% CI, *n* = 27–67. **(B)** Comparison of human CD45^+^ in PB of mice inoculated with either SK-OV-3, OVCAR-5 or patient-derived xenograft BC_038, week 20. 2-way ANOVA, error bars indicate median ± IQR. *n* = 22–26, ns, not significant. **(C)** Total immune cell infiltrate in different tumor models from HIS-NOG-EXL and HIS-NOG, 2-way ANOVA, HIS-NOG + SK-OV-3 *n* = 4, HIS-NOG-EXL + SK-OV-3 *n* = 4, HIS-NOG + OVCAR-5 *n* = 3, HIS-NOG-EXL + OVCAR-5 *n* = 6, HIS-NOG + BC_038 *n* = 8, HIS-NOG-EXL + BC_038 *n* = 5. **(D)** Human leukocytes composition (CD45^+^) in different tumor models in HIS-NOG and HIS-NOG-EXL mice, HIS-NOG + SK-OV-3 *n* = 4, HIS-NOG + OVCAR-5 *n* = 7, HIS-NOG + BC_038 *n* = 4, HIS-NOG-EXL + SK-OV-3 *n* = 4, HIS-NOG-EXL + OVCAR-5 *n* = 12, HIS-NOG-EXL + BC_038 *n* = 5. **(E)** Pie charts of immune subsets in SK-OV-3, OVCAR-5 or BC_038 tumors, Myeloid cells (CD68/CD66b) in CD33+: HIS-NOG + SK-OV-3 *n* = 4, HIS-NOG-EXL + SK-OV-3 *n* = 4, HIS-NOG + OVCAR-5 *n* = 6, HIS-NOG-EXL + OVCAR-5 *n* = 12, HIS-NOG + BC_038 *n* = 3, HIS-NOG-EXL + BC_038 *n* = 4 and DCs (CD303+/CD123+, CD1c+/CD11c+, CD141+/CLEC9A+) in HLA-DR+: HIS-NOG + SK-OV-3 *n* = 4, HIS-NOG-EXL + SK-OV-3 *n* = 4, HIS-NOG + OVCAR-5 *n* = 7, HIS-NOG-EXL + OVCAR-5 *n* = 4, HIS-NOG + BC_038 *n* = 4, HIS-NOG-EXL + BC_038 *n* = 5.

Encouraged by the reasonable numbers of tissue resident pDCs in both NOG strains, we asked whether pDCs could be found in tumors implanted into humanized mice. Hence, two ovarian cancer cell lines and one patient derived breast cancer model were analyzed in parallel in HIS-NOG and HIS-NOG-EXL mice. All tumor models engrafted efficiently into both HIS-NOG strains. SK-OV-3 tumor growth did not differ between the two strains, whereas OVCAR-5 showed a considerably reduced tumor growth in HIS-NOG-EXL (Figure 5A, Supplementary Figure 5A). BC_038 breast tumor growth onset was delayed and at later stages tumor growth accelerated in HIS-NOG-EXL as compared to HIS-NOG (Figure 5A).

To elucidate whether tumors can influence the engrafted human immune system, we analyzed blood samples during tumor growth. Human CD45^+^ immune cell and CD33^+^ myeloid cell frequencies were maintained in peripheral blood independent of tumor growth, with higher myeloid counts in HIS-NOG-EXL as previously described for non-tumor-bearing mice (Figure 5B, Supplementary Figure 5B). Flow cytometric analysis of the tumor immune infiltrate revealed that SK-OV-3 showed the highest frequency of CD45^+^ cells in both mouse strains followed by BC_038 and OVCAR-5. Of note, OVCAR-5 tumors retained their sparse human immune cell infiltrate despite the better humanization of HIS-NOG-EXL mice. In contrast, SK-OV-3 and BC_038 tumors had higher immune infiltration in HIS-NOG-EXL mice (Figure 5C). However, the immune cell composition was clearly influenced by the tumor rather than the HIS mouse strain (Figure 5D). While SK-OV-3 tumors had remarkably high NK cell frequencies, BC_038 and OVCAR-5 showed strong T cell infiltration (Figure 5D).

Analogous, also the myeloid subset distribution was rather shaped by the tumor than the HIS-strain. The three tumor models showed infiltration of human CD68^+^CD66b^−^ TAMs and CD68^−^CD66b^+^ granulocytes, but to different degrees. Whereas, in SK-OV-3 human TAMs were enriched, an equal distribution of granulocytes and TAMs was detected in OVCAR-5 and BC_038 with a higher number of CD66b^−^ CD68^−^ immune cells that might account for immature MDSCs. However, OVCAR-5 and BC_038 tumor-bearing HIS-NOG-EXL mice showed higher TAM infiltration compared to HIS-NOG (Figure 5E). A closer analysis of HLA-DR^+^ myeloid cells revealed presence of human DC subsets in all tumor models albeit at different frequencies (Figure 5E). Human pDCs were primarily detected in OVCAR-5 and BC_038 tumors (Figure 5E, Supplementary Figure 5C).

### pDC Infiltration of Tumors Is Associated With the Presence of pDC-Recruiting Chemokines

We were able to detect pDCs in OVCAR-5 and BC_038, but not in SK-OV-3 tumors. Therefore, we investigated the expression of factors that are associated with pDC function and recruitment. hFLT3-L, hGM-CSF and hIL-3 are well known to correlate with pDC survival and differentiation (39–41). In addition, various migratory cues have been identified to direct pDCs from BM into the blood or diseased tissues including tumor-derived CXCL12 (SDF-1), CXCR3 ligands CXCL9/10 and CCR7 ligands CCL19 and CCL21 (6, 9, 42–47).

**Figure 6:**
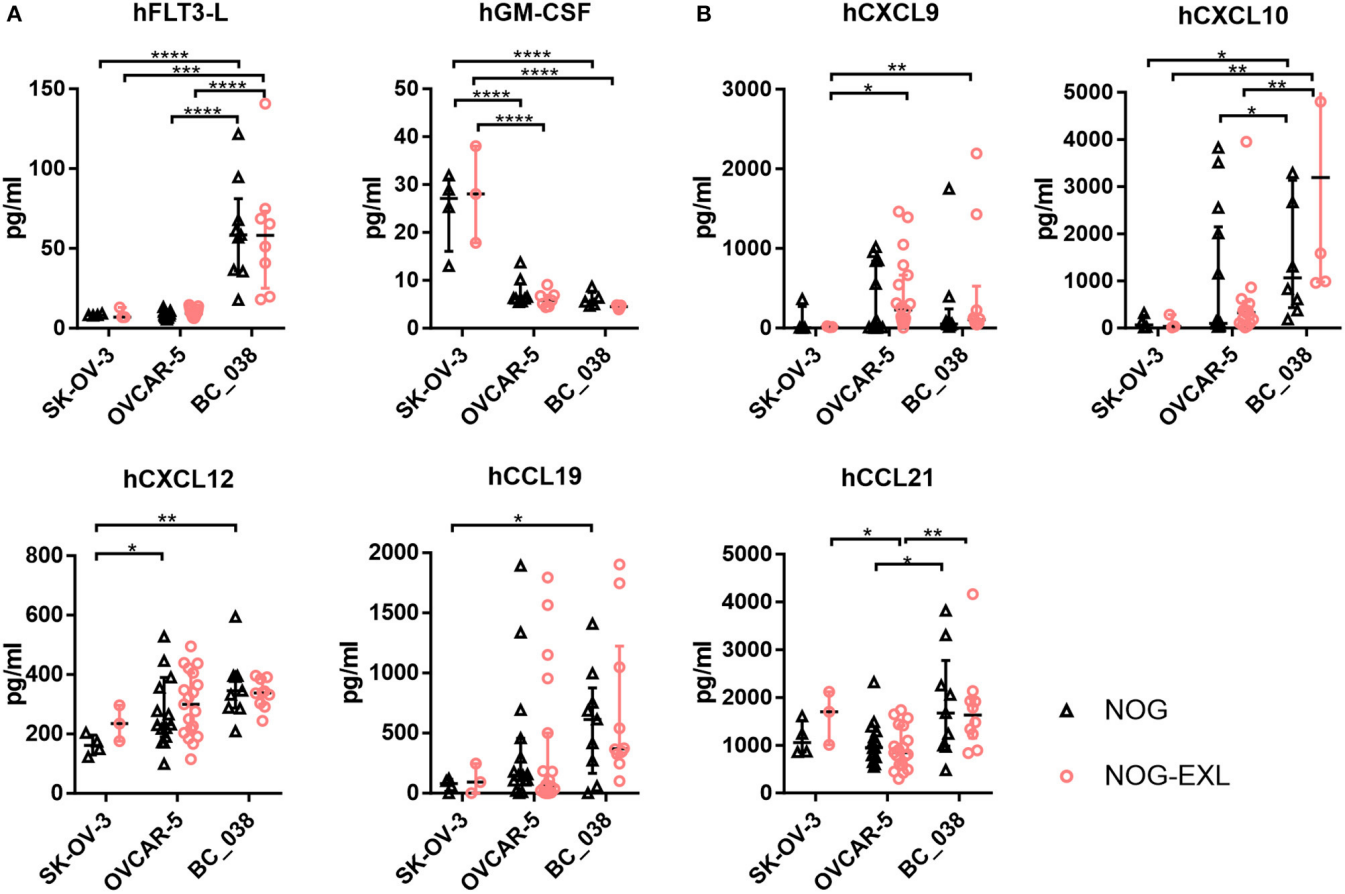
Intratumoral analysis of pDC trafficking and survival associated chemokines and cytokines of different tumors in NOG and NOG-EXL mice. **(A)** Cytokines hFLT-3L, hGM-CSF, and hIL-3 analyzed by Bio-Plex from tumor lysates of humanized mice inoculated with SK-OV-3, OVCAR-5 or BC_038, Kruskal-Wallis ANOVA, error bars indicate median ± IQR, ***p < 0.0005, ****p < 0.0001, error bars indicate median ± IQR, *n* = 3–19. **(B)** Chemokines associated with pDC migration hCXCL9, hCXCL10. hCXCL12, hCCL19, and hCCL21 analyzed by Bio-Plex from tumor lysates of humanized mice inoculated with SK-OV-3, OVCAR-5 or BC_038, Kruskal-Wallis ANOVA, error bars indicate median ± IQR, *p < 0.05; **p < 0.01, ***p < 0.0005, ****p < 0.0001, *n* = 3–19.

Hence, we investigated expression levels of these cytokines and chemokines in tumor lysates of humanized mice bearing SK-OV-3, OVCAR-5 and BC_038 tumors. hGM-CSF and hIL-3 transgene expression in NOG-EXL mice had no effect on the inoculated tumors as their levels were comparable between the two mouse strains (Figure 6). However, we detected striking differences among individual tumor models: SK-OV-3 tumors for example expressed significantly higher levels of hGM-CSF (Figure 6). In contrast, the breast cancer PDX BC_038 showed uniquely high human FLT3-L expression. hIL-3 only showed residual levels barely above the detection limit in all tumors and are therefore not shown. OVCAR-5 and BC_038 had in general a more pronounced and diverse pDC recruiting chemokine profile compared to SK-OV-3 tumors comprising the CXCR3 ligands CXCL9 and CXCL10 as well as CCL19 and CXCL12 (Figure 6). Highest CCL21 expression levels were found in BC_038 (Figure 6). These findings suggest that each tumor generates its specific pDC-recruiting and sustaining network of chemokines and cytokines.

### Intratumoral Stimulation With TLR7/8 Agonist Results in pDCs Activation and IFN-α2 Release

**Figure 7:**
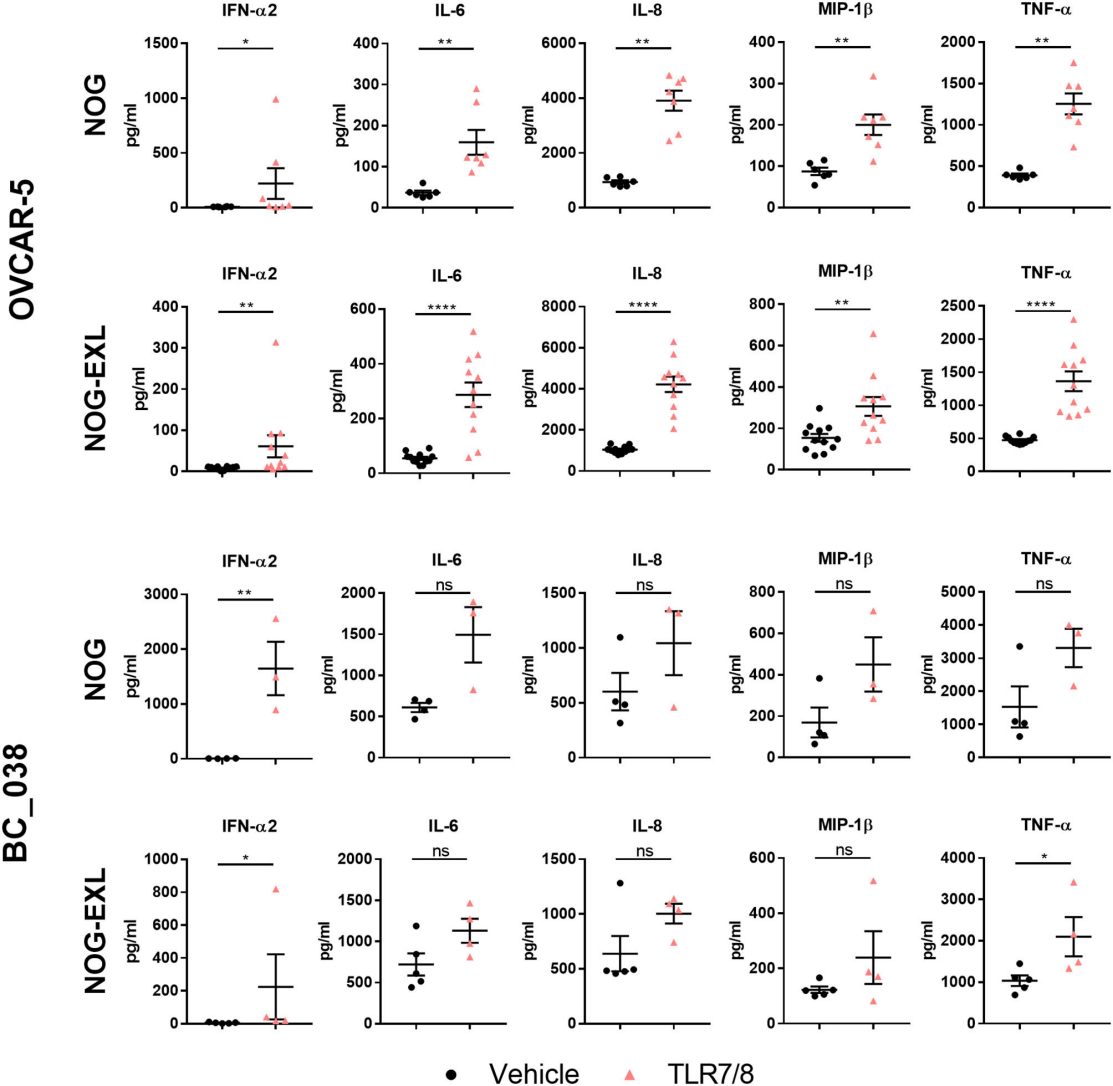
TLR7/8 treatment increases intratumoral cytokine release. Human cytokines analyzed by Multiplex from tumor lysates of humanized mice treated i.t. with TLR7/8 or Vehicle for 4 h in OVCAR-5 and BC_038 tumors; Mann Whitney test; error bars indicate median ± IQR, *p < 0.05; **p < 0.001; ****p < 0.0001; *n* = 4–12.

To further characterize the pDC population in OVCAR-5 and BC_038 we carried out functional analysis of pDCs in these two tumor models transplanted into both NOG strains by injecting the TLR7/8 agonist R848. Notably, tumor-derived pDCs responded to TLR stimulation with upregulation of CD69, CD86, and CD83 after 4 h. Consistently, we also detected increased IFN-α2 secretion in whole tumor lysates (Figure 7). Previous reports demonstrated that TLR7/8 agonist treatment results in secretion of proinflammatory cytokines (13–15, 48–50). Therefore, we profiled other human cytokines in the TME and found elevated levels of IL-6, IL-8, MIP-1β, and TNF-α upon short-term TLR7/8 stimulation (Figure 7). To confirm that cytokine secretion was immune cell dependent, we stimulated OVCAR-5 tumor cells *in vitro* with TLR7/8 and were not able to detect tumor cell line-derived IFN-α2 (data not shown). We were not able to perform a similar experiment using BC_038, which is a primary patient-derived xenograft without an existing cell line.

TLR agonist administration can stimulate dramatic cytokine release in the periphery. Excessive cytokine levels however are also associated with common side effects of immunotherapies. We found that i.t. TLR activation resulted in systemic serum cytokine increase as early as 4 h (Supplementary Figure 6A). For comparison, we injected non-tumor-bearing, humanized mice i.p. with R848 and found relatively higher IFN-α2 levels in serum of both NOG strains compared to i.t injected tumor-bearing mice. This indicates that TLR agonists and the subsequently induced cytokine response are primarily retained within the tumor tissue (Supplementary Figure 6B).

## Discussion

Therapeutic targeting of innate immunity holds great promise for the treatment of immune checkpoint blockade resistant cancer patients, because these patients are likely to have malfunctions in the early, innate immunity-regulated steps of mounting an anti-tumoral immune response or harbor an immune suppressive TME. Targeting of pDCs in the TME could potentially reprogram intratumoral immune cells by triggering the release of a broad type I interferon spectrum shown to reduce tumor growth and activate other immune cells such as NK cells (5, 11, 51). TLR7 and TLR9 agonists are able to activate pDCs and are currently at various stages in clinical development as drug or adjuvants (52, 53). Identification of synergistic combination partners, novel biomarkers or alternative pDC-targeting strategies requires preclinical models that provide sufficient and functional human pDCs in a human TME. In our study, we aimed to find a suitable preclinical, tumor-bearing HIS model taking into account the local tumor milieu and the general quality of human hematopoiesis. Furthermore, we assessed whether improved systemic reconstitution of the human immune system would facilitate a more pronounced tumor-associated pDCs infiltrate to allow *in vivo* functional characterization of this notoriously difficult to study, rare pDCs population.

Despite their limitations, humanized mice are a highly useful and important model system for cancer immunotherapy studies as they provide an *in vivo* testing opportunity for human therapeutic targets that lack a functional mouse homolog. HIS mice come in many different flavors and diverse immune cell reconstitution levels (21). Classical humanized mice, such as BRG, NOG and NSG mainly develop B and T cells and provide a robust model to study tumor interaction with those lymphoid populations e.g., for checkpoint inhibitor or T cell recruitment studies (21, 26, 28, 54–56). However, they lack proper antigen-specific immune responses and suffer from compromised innate immune cell development due to limited cross-reactivity between human and murine cytokines (21, 26, 34). To overcome the latter obstacle, human cytokines e.g., M-CSF, GM-CSF, IL-3, and SCF were introduced using different methods such as hydrodynamic plasmid injection, daily injections of recombinant FLT3-L, genetic transgene or knock-in replacement (28–30, 51, 57, 58). However, also tumor cells can serve as a rich source of human cytokines. That prompted us to ask whether tumor implantation could further boost human myeloid cell reconstitution in tumors transplanted into next generation humanized mouse strains. Therefore, we first performed a comprehensive characterization of two novel, non-tumor-bearing, myeloid enhanced HIS models (HIS-NOG-EXL and HIS-NSG-SGM3), followed by comparison with tumor-bearing HIS-NOG and HIS-NOG-EXL mice to delineate the contributions of tumor and transgene-derived cytokines to human immune cell reconstitution.

We confirmed higher overall engraftment and increased frequency of myeloid cells in peripheral blood of both transgenic mouse strains as compared to non-transgenic HIS-NOG mice (29, 30). Additionally, HIS-NOG-EXL mice showed increased NK cell reconstitution, while NSG-SGM-3 mice are characterized by rather high Treg frequencies and development of granulocytes. Although NOG-EXL and NSG-SGM3 strains harbor the hGM-CSF transgene, we detected 400-fold higher GM-CSF serum levels in NSG-SGM-3 mice that potentially, together with additionally increased G-CSF, favor granulocyte formation over macrophages and DCs. These unusually high GM-CSF levels in HIS-NSG-SGM3 mice may inhibit pDC survival or egress from bone marrow and explain the absence of peripheral pDCs in this strain despite the reported presence of pDC in bone marrow (29, 39, 59). Notably, the pDC inhibitory effect of GM-CSF seems to be dose-dependent in pDCs cultured *in vitro* (60, 61). On the other hand, it was shown that GM-CSF and IL-3 are essential for human pDC viability and differentiation (60). As those two cytokines are systemically expressed in both transgenic mouse strains, albeit to different levels, we hypothesize that GM-CSF levels in HIS-NSG-SGM3 exceed a certain threshold and become inhibitory for pDCs, whereas lower GM-CSF levels in HIS-NOG-EXL promote pDC viability and differentiation. NSG-SGM3 mice also had higher peripheral T cell frequencies and IFN-γ level compared to NOG-EXL. Furthermore, additional inflammatory signals such as IL-6, IL-8, IL-16, MCP-1, TNF-α, CX3CL1, were increased in HIS-NSG-SGM3 mice confirming previous reports (36, 37). Furthermore, the Treg recruiting chemokine CCL22 was significantly elevated. Thus, it is tempting to speculate that the high numbers of immunosuppressive Tregs associated with higher IL-10 levels suppress the activated T cell phenotype. Collectively, the observed hepatosplenomegaly and reduced overall survival suggest that human myeloid cells induce a continuous, smoldering inflammation in HIS-NSG-SGM3 (35–37). This assumption is supported by the notion that depletion of myeloid cells using an anti-CD33 antibody ameliorated these symptoms (36). Another major difference lies in the additional transgene encoding stem cell factor (SCF) in SGM-3 mice that may explain some of the observed differences between NOG-EXL and SGM-3 mice.

Furthermore, the different mouse background may also tune differential immune composition of HIS-NSG-SGM3 compared to HIS-NOG-EXL mice. Consistently, DCs were previously reported in higher numbers in BM of HIS-NOG mice compared to NSG mice (62, 63). HIS-NOG-EXL myeloid cell frequencies are substantially below human levels, although these mice show enhanced myelopoiesis and reconstitute even major subsets of DCs at high frequencies in spleens indicating terminal differentiation and successful egress of these cells from BM (64). Accordingly, also CD14^+^ monocyte count in HIS-NOG-EXL is lower compared to the human situation, while the CD14^+^CD16^+^ non-classical monocyte population is almost absent. Notably, we were able to detect TAMs in SK-OV-3 tumors that express M-CSF, the cytokine responsible for macrophage differentiation and survival (28, 65). In contrast, HIS-NOG-EXL mice had low levels of macrophages in spleens and in general a more immature monocyte phenotype in peripheral blood that may be attributed to the lack of M-CSF (28). Importantly, pDC frequencies in blood and lymphoid organs of HIS-NOG-EXL mice are substantially higher compared to humans (66). This fact enables functional studies of the rare pDC population without further manipulation such as *ex vivo* expansion. The transgene-derived hIL-3 levels that are about 10-fold higher than the transgene-derived hGM-CSF levels (Supplementary Figure 7A), might result in augmented pDC viability in response to activated IL-3R (CD123) signaling.

Based on the important role of pDCs in ovarian and breast cancer, we selected two ovarian cancer cell lines with high and low cytokine producing profile and one breast cancer PDX for detailed human immune analysis in the myeloid enhanced HIS-NOG-EXL as compared to HIS-NOG mice. We compared tumor growth kinetics and found that SK-OV-3 tumor growth is similar in HIS-NOG and HIS-NOG-EXL, whereas OVCAR-5 and BC_038 tumors grew faster or slower in HIS-NOG mice, respectively. GM-CSF and IL-3 are both known to stimulate tumor cell proliferation (67, 68), so their direct effects on tumor growth cannot be excluded. Despite the remarkably altered WBC composition between the different strains, the tumor immune infiltrates were largely comparable between the three different tumor models, albeit striking differences were observed in frequency of individual immune subsets. In contrast to BC_038 and OVCAR-5 tumors, the SK-OV-3 tumor failed to recruit and sustain pDCs.

The molecular mechanism underlying the trafficking of pDCs to the tumor remain largely elusive. Unlike other immune subsets, pDCs can express non-functional chemokine receptors. Under homeostatic conditions pDCs are confined to lymphoid tissues in response to CXCL12 (SDF1)-mediated recruitment via CXCR4 expressed on pDCs (43). However, circulating pDCs express additional chemokine receptors that fail to migrate toward inflammatory stimuli. In pathological tissues pDCs are detectable. Several factors have been described, which may contribute to pDC recruitment into tumors: (i) tumor cells are capable to hijack the CXCL12/CXCR4 axis by producing SDF-1 (9, 46, 69); (ii) pDC maturation in the tumor increases CCR7 or CXCR3 levels and allows these receptors to engage with the migration machinery upon binding to CCL19/CCL21 and CXCL9/CXCL10, respectively (42–44); (iii) Maturation is induced e.g., by extracellular self-DNA bound to HMGB1 protein that prevents its degradation and is recognized by pDCs as TLR ligand (47). High expression levels of CXCL12 were first described in ovarian tumors, later also in various solid tumor entities including breast cancer. CXCL12 was reported to attract pDCs into the tumor environment and to protect them against tumor-induced apoptosis (46, 47, 69, 70). Indeed, BC_038 and OVCAR-5 tumors had higher expression levels of the migratory chemokines CXCL10, CXCL12, and CCL19 (Figure 6) compared to SK-OV-3 and also shared a rather similar cytokine/chemokine profile (Supplementary Figure 7B) and immune cell composition, despite their different origin as PDX and established cancer cell line from a different tumor entity. Moreover, SK-OV-3 tumors expressed strikingly higher GM-CSF levels that may be above the threshold of acquiring pDC-inhibitory function. On the other hand elevated GM-CSF level could benefit SK-OV-3 tumors in shaping their rich TAM-infiltrate along with M-CSF.

Further characterization of tumor-associated pDCs showed upregulation of activation markers and increased IFN-α2 levels upon TLR-agonist treatment. Considering the intratumoral TLR agonist application and the early readout 4 h after agonist injection associated with increased activation marker expression by the pDCs, it is very likely that the observed IFN-α2 increase in tumor lysates originates from pDCs. This is further supported by another study using TLR7/8 agonist stimulated human PBMCs where pDCs were the only producers of IFN-α2 after 4 h of stimulation. Neither T nor B cells responded to TLR treatment while monocytes were strongly activated through the TLR8 agonist and produced TNF-a, IL-6 and IL-1β but not IFN-α2. Furthermore, activated pDCs produced TNF-α and IL-1β (71), confirming findings described in earlier studies (48, 49), which independently showed that pDCs produce not only IFN-α but also TNF-α, IL-6, IL-8, and MIP-1β upon TLR stimulation. The tumor lysate analysis is limited by the lack of analyzing the isolated, pure pDC subset. Purification of sufficient pDC cell numbers from tumors was technically not feasible due to the substantial loss of pDCs during the tumor digestion and immune cell enrichment processes. Therefore, we performed an *in vitro* assay with TLR7/8 agonist treated tumor cell lines as additional control demonstrating no IFN-α2 release. Regarding the other TLR7/8 agonist-induced cytokines we cannot exclude potential contributions from other immune cells such as monocytes. To unequivocally demonstrate that pDC are the predominant IFN-α source, pDC depleting/blocking studies e.g., with the anti-BDCA-2 monoclonal antibody 15B (72–74) or with the BDCA-2-DTR transgenic mice (75) would be necessary.

Thus, our data clearly indicate that local cytokine production by tumor xenografts have a more significant impact on tumor immune infiltrate than the selected humanized mouse strain. Consequently, different tumor-associated immune subsets can potentially be generated in conventional HIS models by selecting the appropriate tumor model. Instead of investing in expensive mice or developing time-consuming new mouse models, screening of the right tumor cell line or PDX model may present a faster and more efficient way to identify tumor models enriched with human pDCs. *In silico* criteria for tumor selection may include the presence of GM-CSF, IL-3 and FLT3-L, ligand expression of chemokine receptors CXCR4, CCR7, and CXCR3 and the absence of very high GM-CSF level. For melanoma models presence of BRAF^V600E^ and absence of NRAS mutations may serve as additional predictors for higher tumor-resident pDC probability (76).

In the current report, we describe an acute setting, where we measured the effects of TLR7/8 agonists 4 h after injection. The next step to be addressed in future studies is to evaluate long-term effects of pDC infiltration as well as IFN-α production on tumor growth and other tumor-resident immune cells. Particularly, we expect that the investigation of the cross talk between activated pDCs and T cells is experimentally challenging in HIS mice considering the uncertainties around mouse MHC restriction and how reconstitution of additional human APC would interfere with T cell priming (77).

As an additional and unexpected finding, to our knowledge we here show for the first time that myeloid enhanced mice have significantly elevated serum IgG levels indicating improved B cell maturation and function. This was surprising because B cells of classical humanized mice have impaired class switch resulting in minimal antigen-specific IgG production (27, 34). Our findings are accompanied by improved LN development especially in HIS-NOG-EXL. Unlike mouse models that were specifically designed for improved LN development, the LNs in our animals had mostly T cells instead of B cells. However, further experiments, e.g., immunization assays, are needed for a more elaborate functional B cell characterization. In summary, HIS-NOG-EXL mice outperform HIS-NOG and HIS-NSG-SGM3 mice in terms of humanization quality.

Our study provides a comprehensive head-to-head comparison of two myeloid enhanced HIS strains using identical humanization protocols including stem cell donors. Those next-generation HIS strains proved to be superior in terms of a more diverse human immune system reconstitution. In particular, the HIS-NOG-EXL mice represent a robust mouse model to study human innate immune cells in homeostasis and in tumor context. In our view, HIS-NOG-EXL provide the clinically most relevant HIS model to study e.g., immunomodulatory drugs due to the enhanced myeloid and NK cell reconstitution, elevated IgG levels and the ability to reconstitute major immune subsets in different tumor models. Additionally, the relatively high proportion of human pDCs in this mouse model allows studying this rare cell population in more detail without any further manipulation in the context of high quality humanization. Although advanced transgenic HIS models clearly showed improved innate immune subset reconstitution in the periphery, this advantage was lost in TME. Our experimental setup delivers a fertile ground to expand the knowledge on how tumors regulate molecular circuits *in vivo* to shape and exploit human immune cells; a knowledge that potentially unravels novel targets for cancer immunotherapy.

## Data Availability Statement

All datasets generated for this study are included in the article/Supplementary Material.

## Ethics Statement

The animal study was reviewed and approved by Regierung von Oberbayern, Az. ROB-55.2-2532.Vet_02-17-19.

## Author Contributions

I-PM, GH, SH, JE, and CR designed experiments. I-PM and CB performed experiments. I-PM, GH, SH, FN, JE, and CR analyzed and interpreted data. CR, SH, and JE wrote the paper together with I-PM and input from all authors. All authors contributed to the article and approved the submitted version.

## Conflict of Interest

I-PM, GH, SH, JE, and CR are current or former Roche employees. CR was employed by Dr. Carola Ries Consulting. The remaining authors declare that the research was conducted in the absence of any commercial or financial relationships that could be construed as a potential conflict of interest.
