# Supplementary figures and images for: The Tumor Milieu Promotes Functional Human Tumor-Resident Plasmacytoid Dendritic Cells in Humanized Mouse Models
Source: Front Immunol. 2020 Sep 8;11:2082. doi: 10.3389/fimmu.2020.02082 (PMC7507800; doi:10.3389/fimmu.2020.02082)

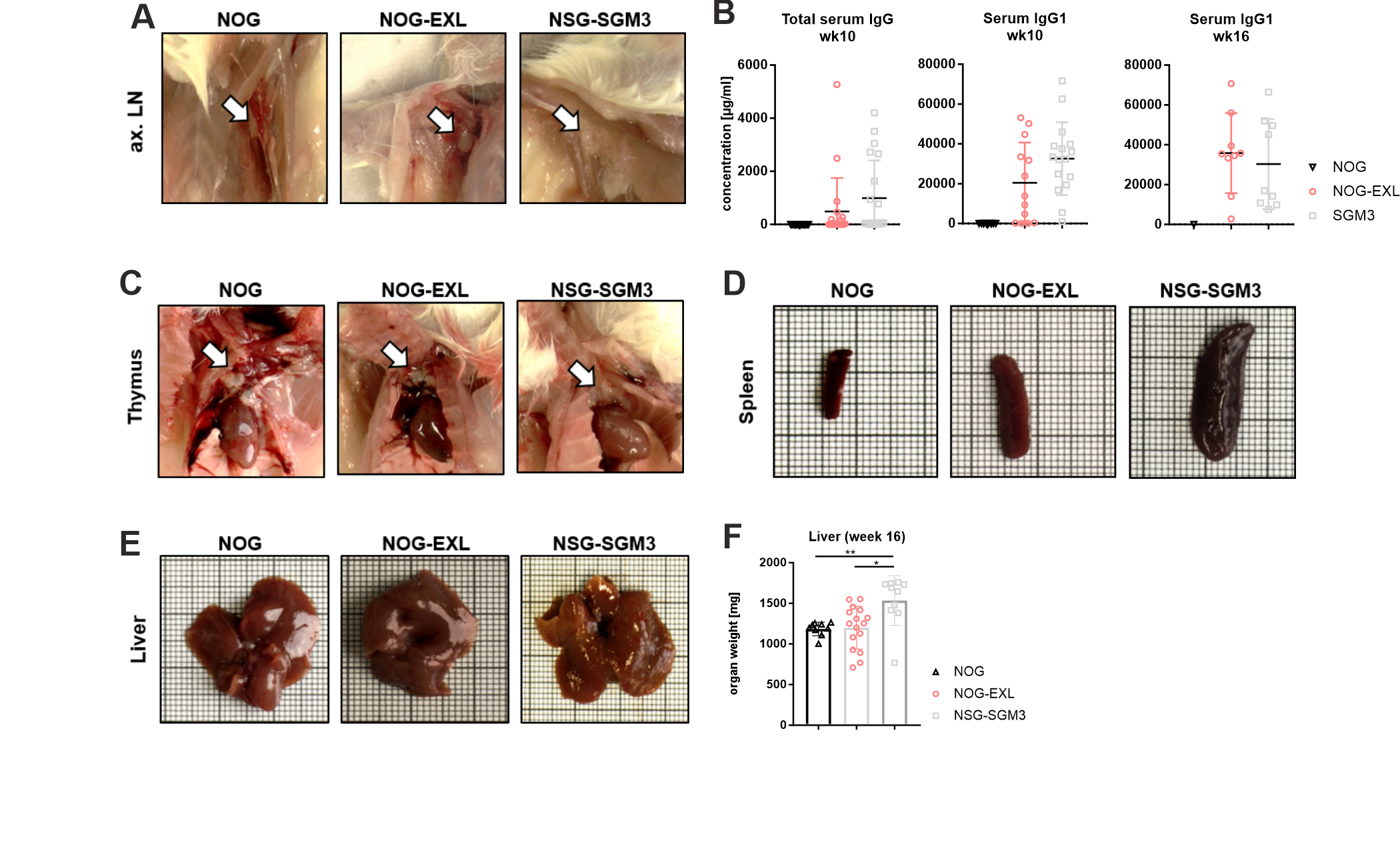

Supplement: Supplementary file 1 [file Image_1.TIF]

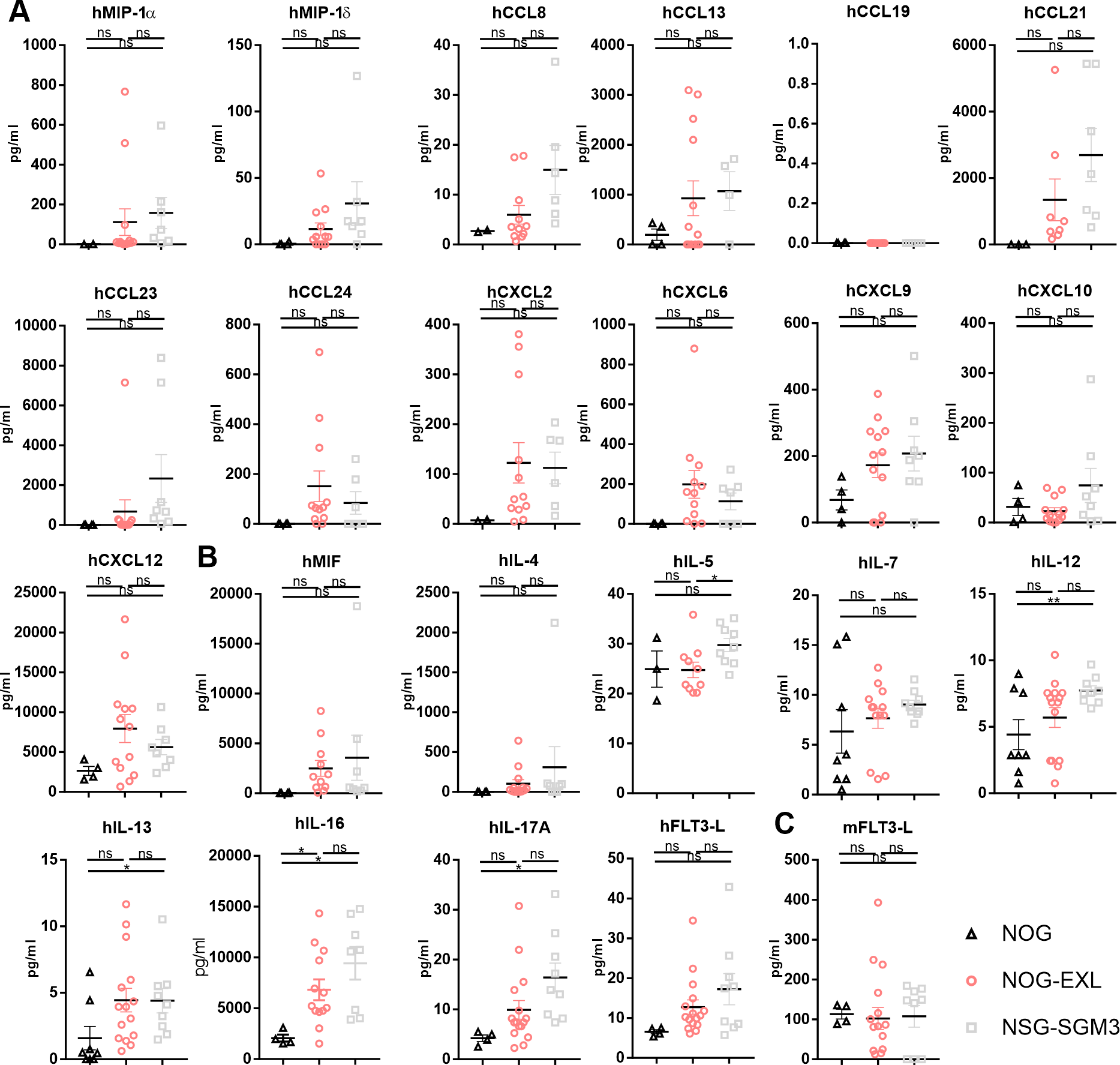

Supplement: Supplementary file 3 [file Image_3.TIF]

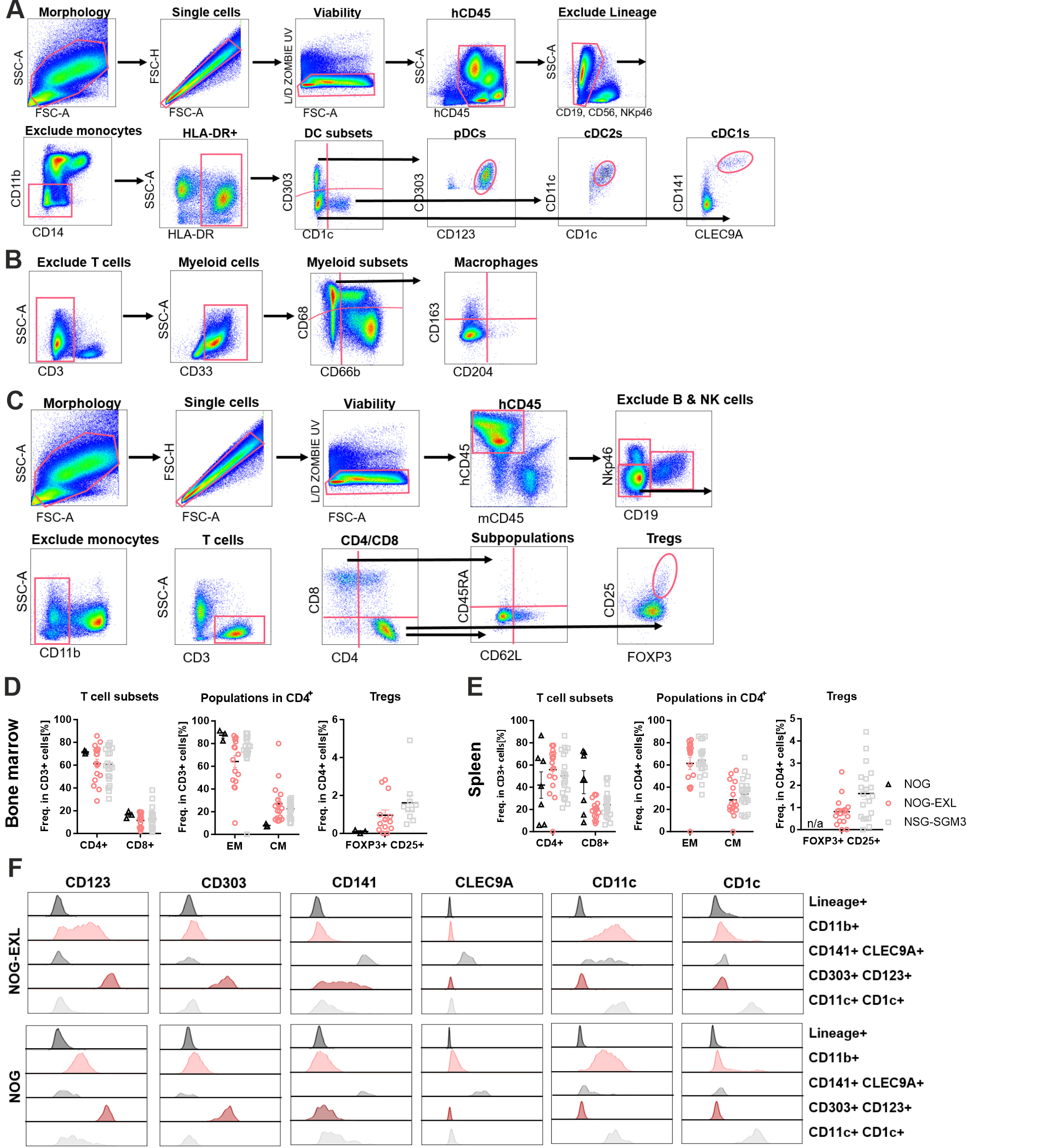

Supplement: Supplementary file 4 [file Image_4.TIF]

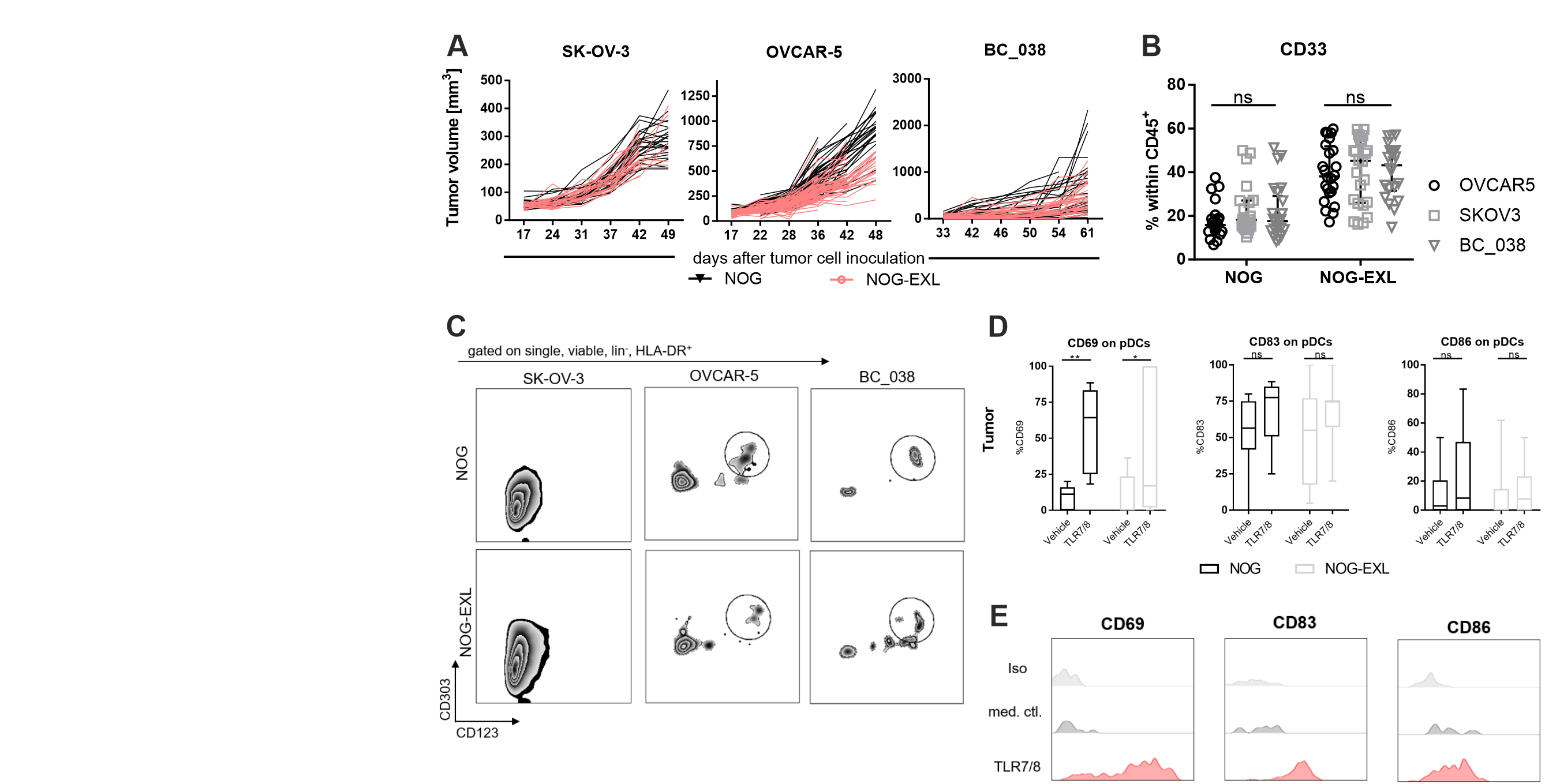

Supplement: Supplementary file 5 [file Image_5.TIF]

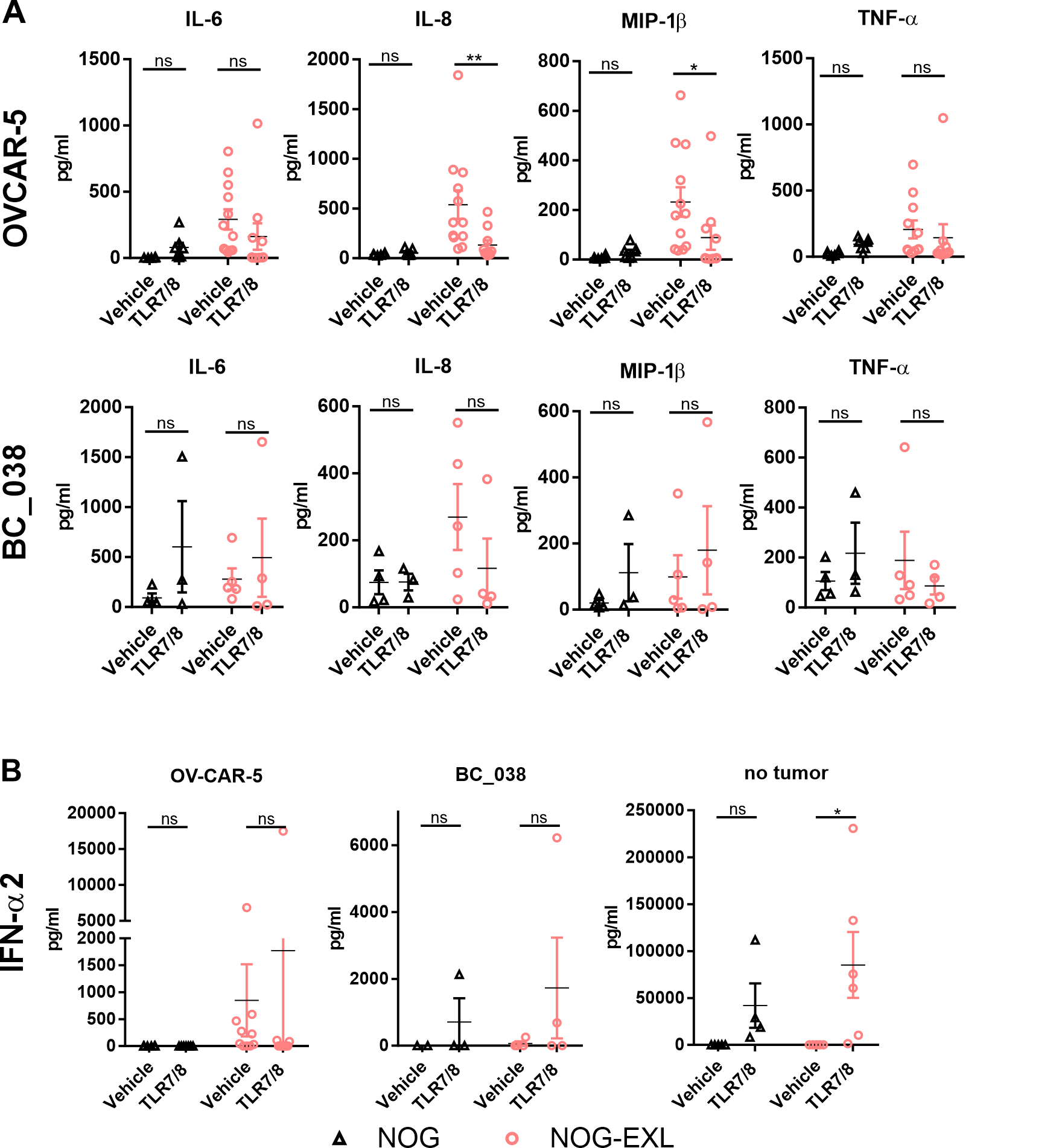

Supplement: Supplementary file 6 [file Image_6.TIF]

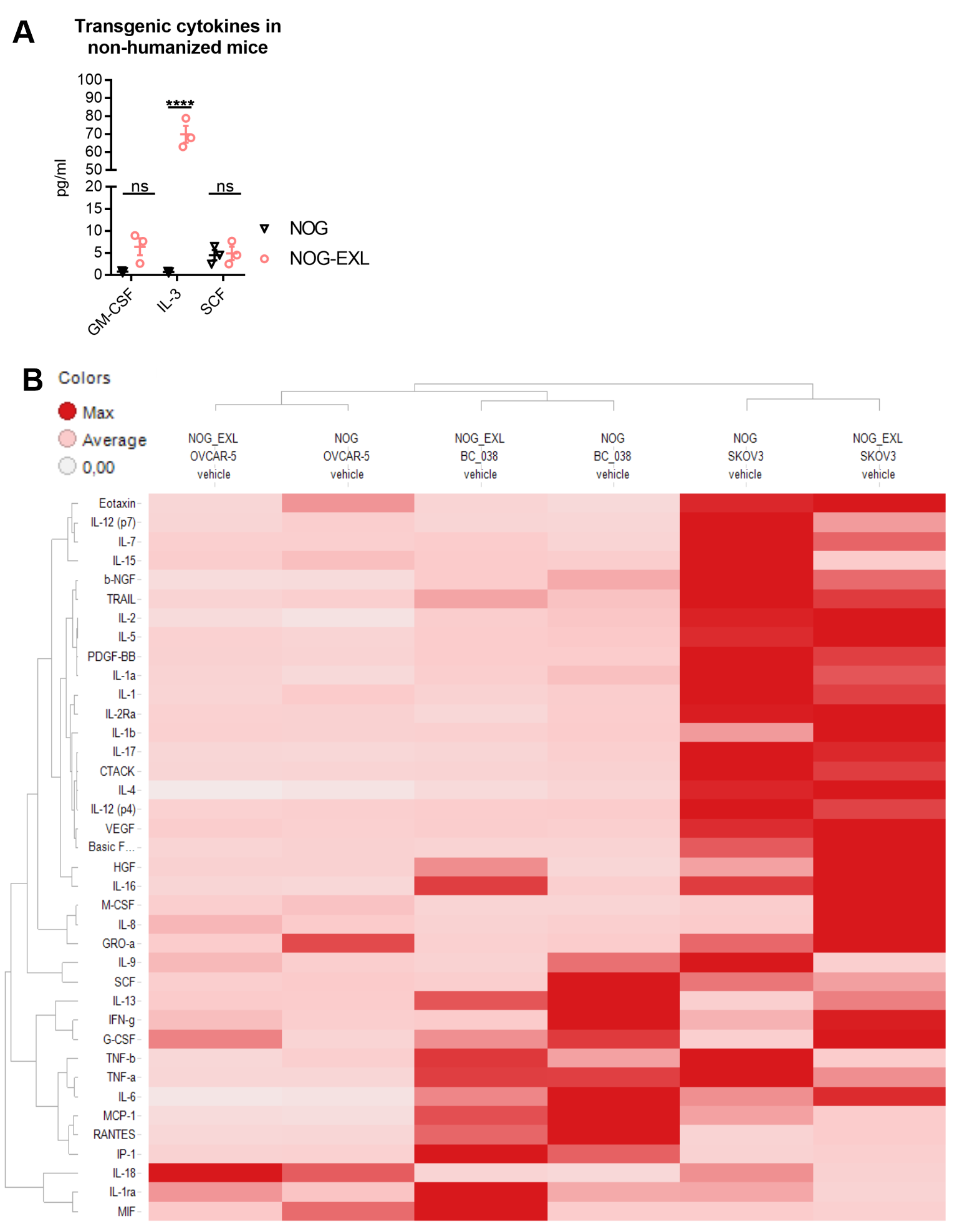

Supplement: Supplementary file 7 [file Image_7.TIF]
