# Supplementary material for: The Tumor Milieu Promotes Functional Human Tumor-Resident Plasmacytoid Dendritic Cells in Humanized Mouse Models
Source: Front Immunol. 2020 Sep 8;11:2082. doi: 10.3389/fimmu.2020.02082 (PMC7507800; doi:10.3389/fimmu.2020.02082)
Supplement: Supplementary file 8 [file Data_Sheet_1.PDF]

# *Supplementary Material*

## 1 Supplementary Figures and Tables

### 1.1 Supplementary Table 1

**FACS antibodies**

| Marker | Clone |
|---|---|
| **CCR7 (CD197)** | 3D12 |
| **CD11b** | ICRF44 |
| **CD11b** | M1/70 |
| **CD11c** | 3.9 |
| **CD123** | 6H6 |
| **CD123** | 7G3 |
| **CD14** | HCD14 |
| **CD14** | M5E2 |
| **CD141** | 1A4 |
| **CD16** | 3G8 |
| **CD163** | Mac2-158 |
| **CD19** | SJ25C1 |
| **CD19** | HIB19 |
| **CD1c** | F10/21A3 |
| **CD20** | 2H7 |
| **CD204** | U23-56 |
| **CD206** | 19.2 |
| **CD25** | 2A3 |
| **CD3** | OKT3 |
| **CD3** | SK7 |
| **CD303** | 201A |

| | |
|---|---|
| **CD33** | HIM3-4 |
| **CD33** | P67.6 |
| **CD4** | SK3 |
| **CD40** | 5C3 |
| **CD45** | HI30 |
| **CD45 RA** | HI100 |
| **CD56** | NCAM16.2 |
| **CD56** | HCD56 |
| **CD62L** | DREG-56 |
| **CD66b** | G10F5 |
| **CD68** | Y1/82A |
| **CD69** | FN50 |
| **CD8** | RPA-T8 |
| **CD83** | HB15e |
| **CD86** | BU63 |
| **CD8a** | SK1 |
| **CLEC9A** | 8F9 |
| **F4/80** | BM8 |
| **FOXP3** | 206D |
| **GZB** | QA16A02 |
| **hCD45** | HI30 |
| **HLA-DR** | G46-6 |
| **mCD45** | 30-F11 |
| **NKp46 (CD335)** | 9 E2 |
| **PD-1** | EH12.1 |
| **TCR a/b** | IP26 |
| **TCR g/d** | B1 |

## 1.2 Supplementary Figure 1

**(A)** Representative images of axial LN. (**B)** Concentration of total human IgG and IgG1 antibodies in serum of humanized mice at week 10 as well as IgG1 in week 16, n= 10/strain, all HIS-NOG mice had values of 0, Kruskal-Wallis ANOVA with Dunn's multiple comparison test, each dot represents an individual mouse, error bars indicate median ± Interquartile range (IQR). (**C)** Representative images of thymus; (**D**) spleen; (**E)** liver. (**F)** Liver weight at termination (week 16): HIS-NSG-SGM3 mice show significantly enlarged liver weights. One-way ANOVA, Tukey's multiple comparison test, each dot represents one individual mouse, error bars indicate mean ± SEM, n= 17 to 23. *, $p < 0.05$; ***, $p < 0.001$; ****, $p < 0.0001$.

## 1.3 Supplementary Figure 2

**(A)** Representative plots and gating strategy of flow cytometry staining in peripheral blood of humanized mice. (**B)** Comparison of percentages of human myeloid cells ($CD33^+$) in human leukocytes ($CD45^+$) at week 12, one-way ANOVA, Tukey's multiple comparison test. *, $p < 0.05$; ***, $p < 0.0005$; ****, $p < 0.0001$; each dot represents one individual mouse, error bars indicate mean ± SEM, n= 10 to 39. (**C)** Myeloid cells ($CD33^+$) in peripheral blood of humanized mice in cts/µl, one-way ANOVA, Tukey's multiple comparison test. *, $p < 0.05$; ***, $p < 0.0005$; ****, $p < 0.0001$; each dot represents one individual mouse, error bars indicate mean ± SEM, n= 10 to 39.

## 1.4 Supplementary Figure 3

**(A)** Human chemokines analyzed by Multiplex from serum of humanized mice, Two-tailed unpaired t-test, *, $p < 0.05$; **, $p < 0.005$; ***, $p < 0.0005$; ****, $p < 0.0001$; each dot represents one individual mouse, error bars indicate mean ± SEM, n= 4 to 16. (**B)** Human cytokines analyzed by Multiplex from serum of humanized mice week 16, Two-tailed unpaired t-test, *, $p < 0.05$; **, $p < 0.005$; ***, $p < 0.0005$; ****, $p < 0.0001$; each dot represents one individual mouse, error bars indicate mean ± SEM, n= 4 to 16. **(C)** Murine cytokine FLT3-L, analyzed by Bio-Plex from serum of humanized mice, Two-tailed unpaired t-test, ns= not significant

## 1.5 Supplementary Figure 4

**A)** Representative dot plots and gating strategy of DC cells in BM but also for spleen, tumor, LN and thymus. (**B)** Representative plots and gating strategy for myeloid cells, first five gates (morphology to exclude lineage) are the same as in A. (**C)** Representative plots and gating strategy for lymphocytes. (**D)** T cell characterization in BM at week 16. EM (Effector memory), CM (Central memory) and (**E)** in spleen. (**F)** Representative histograms from HIS-NOG-EXL and HIS-NOG mice of DCs in spleens, CD303 is exclusively expressed on pDCs ($CD123^+$/ $CD303^+$), CLEC9A on cDC1s ($CD141^+$/$CLEC9A^+$) and CD1c on cDC2s ($CD11c^+$/ $CD1c^+$).

## 1.6 Supplementary Figure 5

**(A)** Tumor volume in $mm^3$ over time of individual humanized mice transplanted with either SK-OV-3, OV-CAR-5 or patient-derived xenograft BC_038. (**B)** Comparison of human $CD33^+$ in $CD45^+$ in peripheral blood, week 20. 2-way ANOVA, Tukey's multiple comparison test, error bars indicate median ± IQR. n= 22 to 26, ns= not significant. (**C)** Representative FACS plots of human pDCs ($CD303^+$ $CD123^+$) in SK-OV-3, OVCAR-5 or BC_038 tumors of HIS-NOG and HIS-NOG-EXL. (**D)** Activation markers (CD69, CD83, CD86) on pDCs in tumor upon stimulation. (**E)** Representative histograms of activation markers with stimulation (TLR7/8) or without stimulation (vehicle), gated on $lin^-$, $HLA\text{-}DR^+$, $pDC^+$.

## 1.7 Supplementary Figure 6

**(A)** Human cytokines analyzed by Multiplex from serum of humanized mice treated i.t. with TLR7/8 or Vehicle for 4h, 2-way ANOVA, uncorrected Fisher's Least Significant Difference (LSD); *, $p < 0.05$; **, $p < 0.001$; ***, $p < 0.0005$; ****, $p < 0.0001$; n= 4 to 12. (**B)** Human IFN-a2 levels analyzed by Bio-Plex from serum of tumor- and non-tumor-bearing humanized mice treated i.t or i.p respectively for 4h.

## 1.8 Supplementary Figure 7

**(A)** Cytokine levels of transgenic cytokines in sera of non-humanized NOG and NOG-EXL mice, 2-way ANOVA, uncorrected Fisher's Least Significant Difference (LSD), ****, $p < 0.0001$, n=3. (**B)** Heat Map showing cytokine levels at baseline of tumors from SK-OV-3, OVCAR-5 and BC_038. Row dendrogram: Clustering method: complete linkage, distance measure: Correlation, ordering weight: Average value, Normalization: None, Scale: Logarithmic. Column dendrogram: Clustering method: Complete linkage, Distance measure: City Block, Ordering weight: Average value, Normalization: None.
